# Supplementary material for: Analysis of the impact of different landing heights and lateralities on lower limb joint load based on statistical parametric mapping
Source: PLoS One. 2025 May 30;20(5):e0324656. doi: 10.1371/journal.pone.0324656 (PMC12124505; doi:10.1371/journal.pone.0324656)
Supplement: Attachment — Submitted filename: PONE-D-25-09215_reviewer_reviewer comments.pdf [file pone.0324656.s002.pdf]

# PLOS ONE

# Analysis of the Impact of Different Landing Heights and Lateralities on Lower Limb Joint Load Based on Statistical Parametric Mapping

--Manuscript Draft--

| | |
|---|---|
| **Manuscript Number:** | PONE-D-25-09215 |
| **Article Type:** | Research Article |
| **Full Title:** | Analysis of the Impact of Different Landing Heights and Lateralities on Lower Limb Joint Load Based on Statistical Parametric Mapping |
| **Short Title:** | Landing Height and Lower Limb Joint Load |
| **Corresponding Author:** | Yuan Gao, PH.D<br>Yanshan University<br>Qinhuangdao, HEHebei Province CHINA |
| **Keywords:** | Statistical parameter mapping; Landing height; Lateralities; Lower limb joint loading |
| **Abstract:** | This study examined changes in lower limb joint loading in rope skipping enthusiasts landing from different heights and on different leg sides. Twenty male participants were assessed using the Qualisys 3D motion capture system and Kistler 3D force plates during landings from 30 cm and 45 cm on both dominant and non-dominant legs. No significant interaction effects between landing side and height were observed, but landing on the non-dominant leg increased ankle joint internal rotation moment. The knee's external abduction moment significantly increased during the 17%-37% landing phase, and the hip's external abduction moment significantly increased during the 86%-100% phase. Higher landing height significantly increased vertical ground reaction force (GRF) during the mid-buffering phase (23%-35%). Landing on the dominant side was linked to an elevated risk of lower limb joint injuries due to increased abduction moments at the hip, external rotation moments at the knee, and internal rotation moments at the ankle. Furthermore, greater landing height correlated with higher vertical GRF, intensifying stress on the lower limbs and increasing injury risk. To reduce the risk of injury, training should focus on increasing strength and control of the non-dominant leg and controlling landing height. |
| **Order of Authors:** | Lian Duan, PH.D |
| | Yuan Gao, PH.D |
| | Feng Gao, PH.D |
| | Yang SUN, M.S |
| | Naiyuan Tian, M.S |
| **Additional Information:** | |
| **Question** | **Response** |
| **Financial Disclosure**<br><br>Enter a financial disclosure statement that describes the sources of funding for the work included in this submission. Review the submission guidelines for detailed requirements. View published research articles from *PLOS ONE* for specific examples.<br><br>This statement is required for submission and **will appear in the published article** if the submission is accepted. Please make | The author(s) received no specific funding for this work. |

*Powered by Editorial Manager® and ProduXion Manager® from Aries Systems Corporation*

| | |
|---|---|
| sure it is accurate.<br><br>**Funded studies**<br>Enter a statement with the following details:<br>• Initials of the authors who received each award<br>• Grant numbers awarded to each author<br>• The full name of each funder<br>• URL of each funder website<br>• Did the sponsors or funders play any role in the study design, data collection and analysis, decision to publish, or preparation of the manuscript?<br><br>Did you receive funding for this work? | |
| **Competing Interests**<br><br>Use the instructions below to enter a competing interest statement for this submission. On behalf of all authors, disclose any competing interests that could be perceived to bias this work—acknowledging all financial support and any other relevant financial or non-financial competing interests.<br><br>This statement is required for submission and **will appear in the published article** if the submission is accepted. Please make sure it is accurate and that any funding sources listed in your Funding Information later in the submission form are also declared in your Financial Disclosure statement.<br><br>View published research articles from *PLOS ONE* for specific examples. | The authors have declared that no competing interests exist |

| | |
|---|---|
| **NO authors have competing interests**<br><br>Enter: *The authors have declared that no competing interests exist.*<br><br>**Authors with competing interests**<br><br>Enter competing interest details beginning with this statement:<br><br>*I have read the journal's policy and the authors of this manuscript have the following competing interests: [insert competing interests here]*<br><br>* typeset | |
| **Ethics Statement**<br><br>Enter an ethics statement for this submission. This statement is required if the study involved:<br><br>• Human participants<br>• Human specimens or tissue<br>• Vertebrate animals or cephalopods<br>• Vertebrate embryos or tissues<br>• Field research<br><br>Write "N/A" if the submission does not require an ethics statement.<br><br>General guidance is provided below. Consult the submission guidelines for detailed instructions. **Make sure that all information entered here is included in the Methods section of the manuscript.** | Human participants<br>Approving Authority: Ethics Committee of Qinhuangdao First Hospital<br>Approval Number: 2024Y006<br>Form of approval: Written |

Powered by Editorial Manager® and ProduXion Manager® from Aries Systems Corporation

| | |
|---|---|
| **Format for specific study types**<br><br>**Human Subject Research (involving human participants and/or tissue)**<br>• Give the name of the institutional review board or ethics committee that approved the study<br>• Include the approval number and/or a statement indicating approval of this research<br>• Indicate the form of consent obtained (written/oral) or the reason that consent was not obtained (e.g. the data were analyzed anonymously)<br><br>**Animal Research (involving vertebrate animals, embryos or tissues)**<br>• Provide the name of the Institutional Animal Care and Use Committee (IACUC) or other relevant ethics board that reviewed the study protocol, and indicate whether they approved this research or granted a formal waiver of ethical approval<br>• Include an approval number if one was obtained<br>• If the study involved *non-human primates*, add *additional details* about animal welfare and steps taken to ameliorate suffering<br>• If anesthesia, euthanasia, or any kind of animal sacrifice is part of the study, include briefly which substances and/or methods were applied<br><br>**Field Research**<br><br>Include the following details if this study involves the collection of plant, animal, or other materials from a natural setting:<br>• Field permit number<br>• Name of the institution or relevant body that granted permission | |
| **Data Availability**<br><br>Authors are required to make all data underlying the findings described fully available, without restriction, and from the time of publication. PLOS allows rare exceptions to address legal and ethical concerns. See the PLOS Data Policy and FAQ for detailed information. | Yes - all data are fully available without restriction |

| | |
|---|---|
| A Data Availability Statement describing where the data can be found is required at submission. Your answers to this question constitute the Data Availability Statement and **will be published in the article**, if accepted.<br><br>**Important:** Stating 'data available on request from the author' is not sufficient. If your data are only available upon request, select 'No' for the first question and explain your exceptional situation in the text box.<br><br>Do the authors confirm that all data underlying the findings described in their manuscript are fully available without restriction? | |
| **Describe where the data may be found in full sentences. If you are copying our sample text, replace any instances of XXX with the appropriate details.**<br><br>• If the data are **held or will be held in a public repository**, include URLs, accession numbers or DOIs. If this information will only be available after acceptance, indicate this by ticking the box below. For example: *All XXX files are available from the XXX database (accession number(s) XXX, XXX.).*<br>• If the data are all contained **within the manuscript and/or Supporting Information files**, enter the following: *All relevant data are within the manuscript and its Supporting Information files.*<br>• If neither of these applies but you are able to provide **details of access elsewhere**, with or without limitations, please do so. For example:<br><br>*Data cannot be shared publicly because of [XXX]. Data are available from the XXX Institutional Data Access / Ethics Committee (contact via XXX) for researchers who meet the criteria for access to confidential data.*<br><br>*The data underlying the results presented in the study are available from (include the name of the third party* | All relevant data are within the manuscript and its Supporting Information files. |

| | |
|---|---|
| *and contact information or URL).*<br>• This text is appropriate if the data are owned by a third party and authors do not have permission to share the data.<br><br>* typeset | |
| Additional data availability information: | Tick here if your circumstances are not covered by the questions above and you need the journal's help to make your data available.; Tick here if the URLs/accession numbers/DOIs will be available only after acceptance of the manuscript for publication so that we can ensure their inclusion before publication. |

Powered by Editorial Manager® and ProduXion Manager® from Aries Systems Corporation

Manuscript Click here to access/download;Manuscript;Manuscript .docx

# Analysis of the Impact of Different Landing Heights and Lateralities on Lower Limb Joint Load Based on Statistical Parametric Mapping

Main text word count **2304**

## Abstract

This study examined changes in lower limb joint loading in rope skipping enthusiasts landing from different heights and on different leg sides. Twenty male participants were assessed using the Qualisys 3D motion capture system and Kistler 3D force plates during landings from 30 cm and 45 cm on both dominant and non-dominant legs. No significant interaction effects between landing side and height were observed, but landing on the non-dominant leg increased ankle joint internal rotation moment. The knee's external abduction moment significantly increased during the 17%-37% landing phase, and the hip's external abduction moment significantly increased during the 86%-100% phase. Higher landing height significantly increased vertical ground reaction force (GRF) during the mid-buffering phase (23%-35%). Landing on the dominant side was linked to an elevated risk of lower limb joint injuries due to increased abduction moments at the hip, external rotation moments at the knee, and internal rotation moments at the ankle. Furthermore, greater landing height correlated with higher vertical GRF, intensifying stress on the lower limbs and increasing injury risk. To reduce the risk of injury, training should focus on increasing strength and control of the non-dominant leg and controlling landing height.



**Keywords:** Statistical parameter mapping; Landing height; Lateralities; Lower limb joint loading

# 1. Introduction

In recent years, rope skipping has emerged as a widely adopted exercise modality, owing to its low cost, ease of learning, and cardiovascular benefits. Its effectiveness in improving both aerobic fitness and coordination has made it a favored exercise among fitness enthusiasts and recreational athletes alike [1]. Freestyle rope skipping, known for its diverse techniques and visually engaging performances, has gained widespread popularity. However, the complexity of these movements elevates the risk of injury, particularly during the landing phase. Recent studies highlight a notably high incidence of lower limb injuries (87.5%), with knee and ankle joints being most frequently affected, comprising approximately 80% of all injury cases [2-4]. Despite the human body's inherent structural symmetry, functional asymmetry, or laterality, is well-documented in lower limb biomechanics [5-6]. Excessive lateral laterality has been identified as a potential risk factor for non-contact joint injuries [7-8]. However, most rope skipping research assumes limb symmetry, primarily analyzing data from a single limb to assess landing-related injury risks [9-12]. Consequently, there is limited understanding of the biomechanical differences between dominant and non-dominant limbs during single-leg landing in rope skipping, highlighting a critical gap in the literature.Statistical Parametric Mapping (SPM), based on random field theory, utilizes topological analysis to examine dynamic changes and interrelationships in time series data, enhancing the robustness of biomechanical conclusions, as indicated by Wattananon et al. [ 13 ] and Pataky et al. [ 14 - 16 ]. While traditional

zero-dimensional statistical methods can identify feature differences, they often fail to capture dynamic patterns in time series data, as highlighted by previous studies [16-18]. To address these limitations, this study utilizes SPM to analyze the biomechanical effects of landing heights and leg lateralities on lower limb joint loading, offering evidence-based insights for injury prevention and performance optimization in rope skipping.

# 2. Materials and methods

## 2.1 Participants

Study subjects were recruited from 15 April 2024 to 23 April 2024, followed by a 12-day experiment to collect the data required for the study. A total of 20 male rope-skipping enthusiasts participated in this study (age: 19.75 ± 0.86 years, height: 181.24 ± 4.62 cm, weight：72.21± 7.66 kg, BMI: 22.06 ± 2.94 kg/m$^2$).

All participants were free from significant sports injuries, neurological disorders, or visual impairments in the past six months. In accordance with the ethical standards of the Declaration of Helsinki, they were fully briefed on the aims, procedures and requirements of the study, informed consent was obtained in writing, and consent was approved by the Ethics Committee of the First Hospital of Qinhuangdao.

The determination of the dominant side was conducted through a kicking test, where participants were asked to kick with both feet. The foot that achieved the greater kicking distance was identified as the dominant side [7, 19].

## 2.2 Single-leg landing test

Participants were instructed to place their hands on their hips and stand 5 cm from the

force platform, with jumping boxes positioned at heights of 30 cm and 45 cm. Upon receiving a "start" command, participants lifted their test leg and held it in the air. They then leaned forward and descended by inertia, landing on the test foot to maintain posture and stability [2, 20]. Participants were required to focus visually on a target displayed on a blank wall in front of them throughout the test. Trials were repeated if participants touched the ground with the non-test leg or used their arms for balance (Fig. 1) [21].

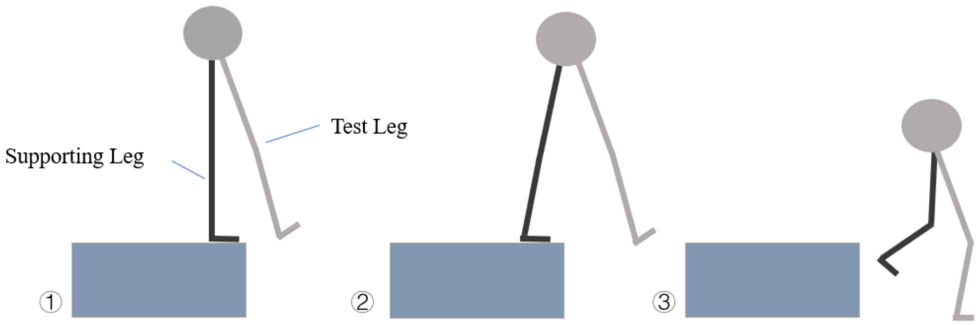


**Fig.1.** Single leg drop test manoeuvre

2.3 Data Collection

During trials, participants wore study-specific tights, and after warming up, 20 infrared reflective markers were placed on anatomical landmarks according to the CAST lower limb model. Following the collection of static model data, eight markers were removed from the medial and lateral sides of the knee and ankle joints. Participants performed at least three complete movements during trials, and kinematic and kinetic data were synchronously captured using the Qualisys 3D motion capture

system (8 cameras, 200 Hz) and a Kistler 3D force platform (2000 Hz) during landing (Fig. 2).

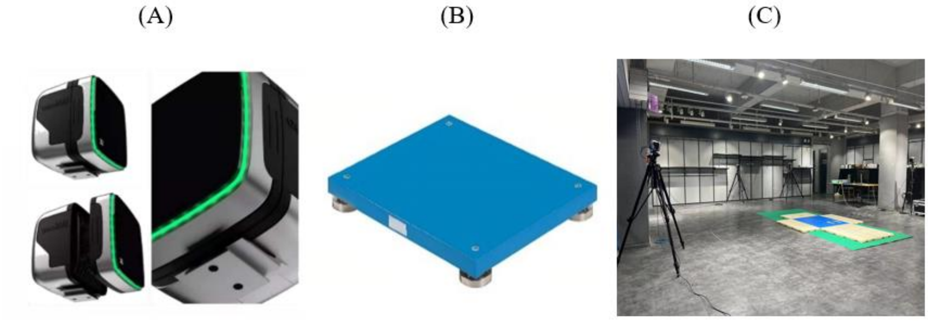


**Fig. 2.** Qualisys 3D Motion Capture System and Kistler 3D Force Stage

Note: The test apparatus included: a Qualisys 3D motion capture system to capture motion in space (A), a Kistler 3D force table to measure changes in force (B), and an experimental test site (C).

## 2.4 Data Processing

In this study, the landing cushion phase ( the moment of contact of the test leg on the ground up to the maximum angle of knee flexion of the supporting leg) of the drop jump movement was recorded. The raw data were pre-processed using the QTM software supplied with the Qualisys system, and hip, thigh, calf, and foot coordinate systems were established based on the CAST lower limb model, using infrared reflective markers to create a simulation of the subject's lower limb. Kinematic data were subsequently imported into Visual 3D software for further processing. A fourth-order Butterworth low-pass filter with a cutoff frequency of 12 Hz was applied to smooth the data, as described by Kristianslund et al. [22], Lenz et al. [23] and

Monfort-Torres et al. [20]. Joint moments for ankle plantarflexion, knee flexion, and hip flexion (N•m•kg) were defined as positive (+) values, while ankle dorsiflexion, knee extension, and hip extension moments were defined as negative (−) values. Ground reaction forces (N) and joint moments (N•m•kg) Lenz et al. [23] were calculated through inverse dynamics (Fig. 3).

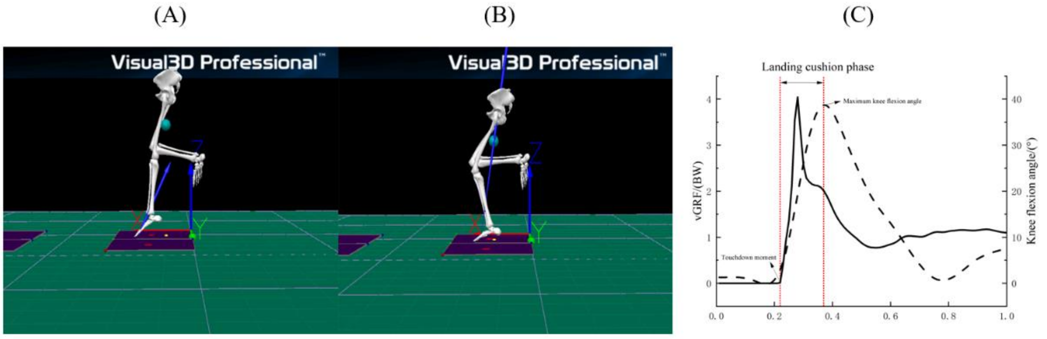


**Fig. 3.** Landed buffer phase

Note: The landing cushioning step is defined as the moment from the initial foot contact with the force platform to the maximum knee flexion angle, A: the moment from the initial foot contact with the force platform, B: the moment from the maximum knee flexion angle, C: the division of the landing cushioning phase.

## 2.5 Statistical Analysis

This study analyzes biomechanical indicators related to lower limb joint loads during the landing cushioning phase, defined as the period from initial ground contact by the supporting leg to maximum knee flexion. Joint moments were normalized to 101 data points across the landing phase for precise temporal resolution. Mean value curves were generated to compare dynamic variations in joint load related parameters. A

two-way ANOVA is employed to examines the effects of side and landing height on peak vertical GRF and 3D joint moments during the cushioning phase, with normality and homogeneity of variance verified. SPM{F} analysis assesses side and landing height effects on vertical GRF and 3D joint moment time series. Statistical significance is set at $P<0.05$. Analyses are performed with SPSS 23.0 and Origin 2022.

# 3. Results

Two-way ANOVA and SPM{F} results showed no significant interaction effects ($P > 0.05$) between side and height.

## 3.1 Ground reaction forces in the landing cushioning phase

The two-way ANOVA results indicated that the peak vertical GRF during the landing cushioning phase was significantly greater for the 45 cm drop height (4.32 BW) compared to the 30 cm drop height (3.48 BW), independent of the side factor, with statistical significance denoted by $p < 0.001$ and $F = 30.914$ (Table 1).

**Table 1.** Comparison of vertical GRF peaks during the landing cushion phase for different side groups and drop heights（BW）

| | D30 | D45 |
|---|---|---|
| Non-dominant | 3.47±0.48[#] | 4.31±0.64 |
| Dominant | 3.49±0.58 | 4.34±0.67 |

Note: Mean (Standard difference), [*]:There was a significant difference between D30 and D45.

The SPM{F} analysis revealed that during the 23% to 35% of the landing cushioning phase, the vertical GRF for the 45 cm drop height was significantly greater than that

for the 30 cm drop height, regardless of the side factor, with P = 0.004 and F = 8.927 (Fig. 4).

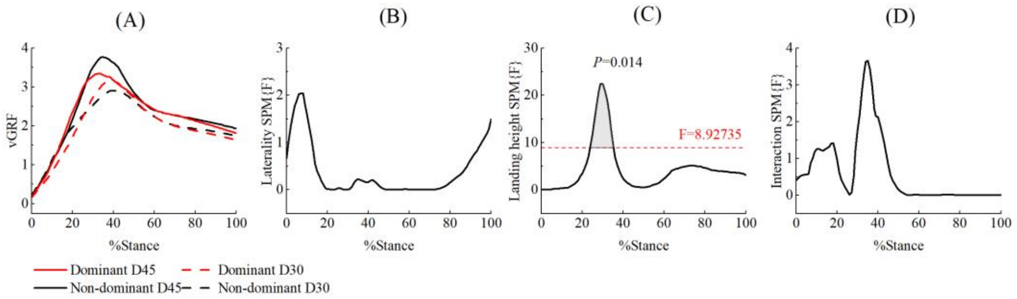


**Fig. 4.** Comparison of vertical GRF SPM{F} in the landing cushion phase for different side and drop heights.

Note: Mean ensemble curves for vGRF (A) and their respective SPM{F} outputs (B, C, D) from 0 to 100% of stance phase. The dashed horizontal line on the SPM{F} outputs indicates the critical threshold boundary. The grey, shaded regions indicates the significant supra-threshold clusters representing statistically significant differences.

## 3.2 Lower limb kinetic characteristics during the landing cushioning phase

The results of the two-way ANOVA indicate that during the landing cushioning phase, the peak dorsiflexion moment of the ankle joint (0.99 N•m•kg$^{-1}$ vs. 1.64 N•m•kg$^{-1}$, P = 0.01, F = 6.901), the peak internal rotation moment of the ankle joint (0.11 N•m•kg$^{-1}$ vs. 0.37 N•m•kg$^{-1}$, P < 0.001, F = 47.348), the peak abduction moment of the knee joint (0.36 N•m•kg$^{-1}$ vs. 0.88 N•m•kg$^{-1}$, P = 0.006, F = 7.834), and the peak external rotation moment of the knee joint (0.19 N•m•kg$^{-1}$ vs. -0.41 N•m•kg$^{-1}$, P < 0.001, F = 58.042), are significantly greater on the non-dominant side compared to the dominant side. In addition, irrespective of the side factor, the peak flexion moment of

the knee joint when landing from a 45 cm height is significantly greater (2.66 N•m•kg$^{-1}$ vs. 2.03 N•m•kg$^{-1}$, P = 0.003, F = 9.132) than when landing from a 30 cm height (Table 2).

**Table 2.** Comparison of the peak moments of the lower limb joints during the landing cushioning phase by side and height of fall.

| | Dominant | | Non-dominant | |
|---|---|---|---|---|
| Peak moment （N·m·kg$^{-1}$） | D30 | D45 | D30 | D45 |
| Ankle dorsiflexion | 0.95±1.39* | 1.04±1.51 | 1.65±0.52 | 1.62±0.49 |
| Ankle valgus | 0.46±0.36 | 0.60±0.45 | 0.49±0.41 | 0.52±0.34 |
| Ankle internal rotation | 0.11±0.15* | 0.11±0.18 | 0.36±0.17 | 0.38±0.17 |
| Knee flexion | 1.90±1.12# | 2.42±1.15 | 2.16±0.65 | 2.89±0.72 |
| Knee valgus | 0.20±0.71* | 0.23±0.98 | 0.90±0.56 | 0.85±0.96 |
| Knee internal rotation | 0.22±0.33* | 0.17±0.47 | -0.30±0.22 | -0.51±0.35 |
| Hip flexion | 0.52±2.21 | 0.74±3.17 | 0.57±1.72 | 2.05±2.14 |
| Hip valgus | 1.79±1.47 | 1.80±1.44 | 1.93±0.49 | 2.13±0.47 |
| Hip internal rotation | 0.61±0.31 | 0.64±0.57 | 0.78±0.30 | 0.83±0.46 |

Note: Mean (Standard difference), *: Significant difference between dominant and non-dominant side, #: There was a significant difference between D30 and D45.

The SPM{F} analysis indicates that during the 86% to 100% of the landing cushioning phase, the peak hip abduction moment is significantly greater on the non-dominant side compared to the dominant side ((F = 8.084, P = 0.022), irrespective of the height factor (Fig. 5). Similarly, during the 17% to 37% of the landing cushioning phase, the peak abduction moment of the knee joint is significantly greater on the non-dominant side ((F = 7.167, P = 0.026), independent of height factor (Fig. 6). Throughout the entire landing cushioning phase, the ankle

internal rotation moment on the non-dominant side is significantly greater than on the dominant side (F = 8.127, P < 0.001) ( Fig. 7). In addition, during the 10% to 38% of the landing cushioning phase, the knee joint flexion moment is significantly greater for a 45 cm landing (F = 8.350, P < 0.001) than for a 30 cm landing, regardless of the side factor (Fig. 6).

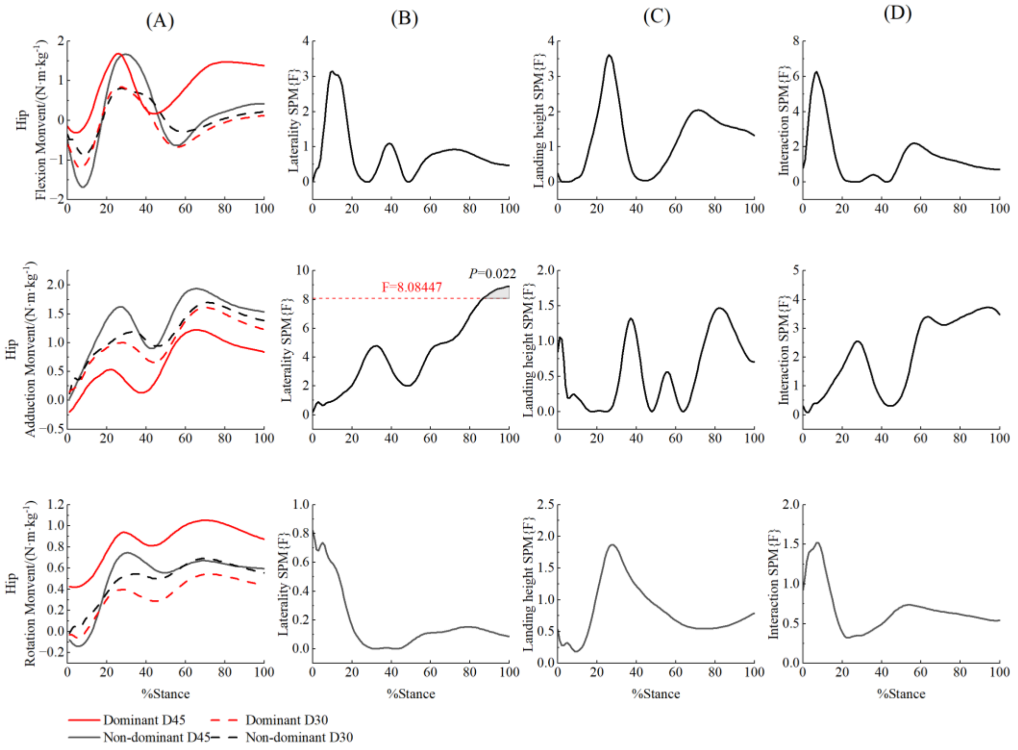


**Fig. 5.** Comparison of hip moments SPM{F} during the landing cushion phase for different side differences and drop heights.

Note: Mean ensemble curves for 3D hip joint moment (A) and their respective SPM{F} outputs (B, C, D) from 0 to 100% of stance phase. The dashed horizontal line on the SPM{F} outputs indicates the critical threshold boundary. The grey, shaded regions indicates the significant supra-threshold clusters representing statistically significant differences. Positive sagittal, frontal, and transverse plane knee joint kinetics represent internally applied knee

extension/adduction/internal rotation moments.

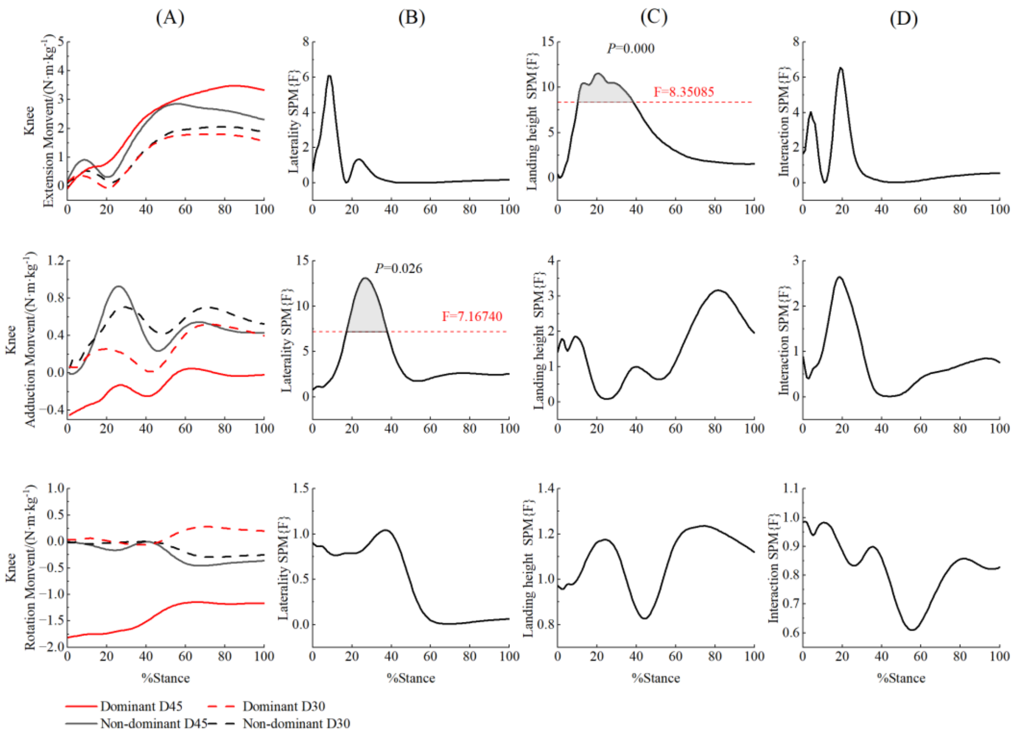


**Fig. 6.** Comparison of knee moments SPM{F} during the landing cushion phase for different side and drop heights.

Note: Mean ensemble curves for 3D knee joint moment (A) and their respective SPM{F} outputs (B, C, D) from 0 to 100% of stance phase. The dashed horizontal line on the SPM{F} outputs indicates the critical threshold boundary. The grey, shaded regions indicates the significant supra-threshold clusters representing statistically significant differences. Positive sagittal, frontal, and transverse plane knee joint kinetics represent internally applied knee extension/adduction/internal rotation moments.

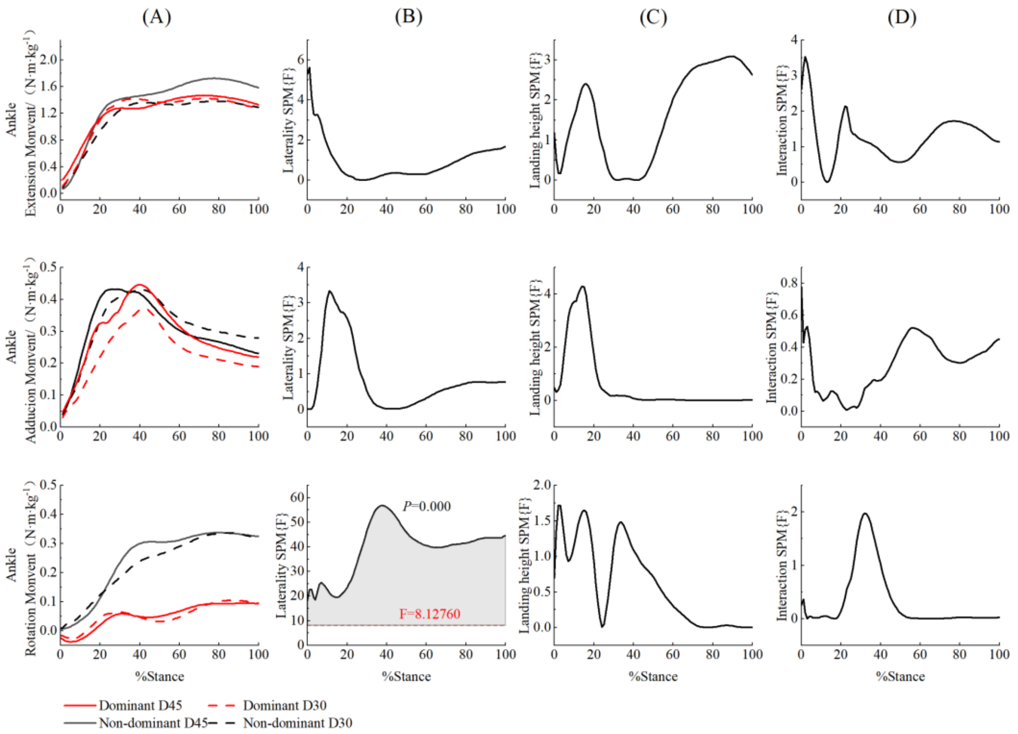


**Fig. 7.** Comparison of ankle moment SPM{F} during the landing cushioning phase for different side and drop heights.

Note: Mean ensemble curves for 3D ankle joint moment (A) and their respective SPM{F} outputs (B, C, D) from 0 to 100% of stance phase. The dashed horizontal line on the SPM{F} outputs indicates the critical threshold boundary. The grey, shaded regions indicates the significant supra-threshold clusters representing statistically significant differences. Positive sagittal, frontal, and transverse plane knee joint kinetics represent internally applied knee extension/adduction/internal rotation moments.

# 4. Discussion and Analysis

4.1 Comparative analysis of side differences during the landing cushioning phase

Two-way ANOVA showed no significant difference in peak 3D hip moments regarding side or height, but SPM{F} revealed higher hip abduction moments on the non-dominant side, reflecting the need for stability during maximum knee flexion and impact absorption [19]. The hip joint stabilizes the coronal plane by increasing abduction moments, working in tandem with precise muscle control and compensatory movements to ensure balance [19, 21]. During the landing cushioning phase, the non-dominant leg exhibits a more pronounced increase in abduction moment to compensate for uneven strength and stabilize overall posture. Excessive hip abduction can shift the center of pressure outward, leading to compensatory changes in adjacent joints, increasing knee valgus and ACL injury risk [101010[101010]10, 24-26]. The larger hip abduction moment in the non-dominant leg during the landing cushioning phase may impact the knee joint within the kinetic chain. Additionally, while SPM{F} analysis showed a significant increase in the non-dominant leg's hip abduction moment, no significant difference in external rotation moment was observed, likely due to the conservative significance threshold used in zero-dimensional statistical analyses to prevent Type I errors [[18]]. Previous research has shown that zero-dimensional models have limitations in deriving probabilistic inferences from one-dimensional dynamic data [15-[17]17]. Although one-dimensional analysis may miss variations in knee joint external rotation, zero-dimensional models might still reveal significant differences. The SPM{F} results show that the non-dominant ankle exhibits a higher internal rotation moment during landing. This is likely due to the ankle's complex anatomy, which is inherently more prone to

deformation upon impact. To compensate, the body increases the internal rotation moment for stability. Furthermore, the non-dominant side, with relatively weaker muscle strength and less precise neural control, may require greater plantarflexion and internal rotation to maintain landing stability [9].

In conclusion, this study highlights the need for targeted strength training for the non-dominant lower limb to improve stability and reduce injury risk. Standardizing techniques and incorporating muscle-strengthening exercises are crucial for optimizing impact absorption and minimizing joint loading, thereby protecting the joints from injury.

### 4.2 Comparative analysis of different heights during the landing cushioning phase

Both two-way ANOVA and SPM{F} analyses confirm that a 45 cm jump height generates significantly higher vertical GRF than a 30 cm jump. This aligns with physical principles: greater landing height increases vertical velocity with constant mass, requiring a higher impulse to counter momentum change. Due to the body's limited impact absorption capacity, excess force translates into higher GRF, amplifying landing impact. This finding supports Ali et al. [27], revealing a significant increase in knee flexion moment during landing from 45 cm compared to 30 cm. Previous studies have established that the knee joint, as the primary weight-bearing and energy-absorbing joint, plays a crucial role in impact force absorption during the cushioning phase [2, 28]. Accordingly, our study shows that higher landing heights result in increased impact forces, placing greater load on the

knee for absorption and cushioning [29]. This is consistent with Leppänen et al. [30], who found a significant positive correlation between knee flexion moments and ACL tension, suggesting a potential increase in ACL injury risk with higher knee flexion during landing [2, 19]. To reduce injury risk, rope jumping participants should control landing heights and gradually increase them during training. Complementing this with strength and coordination exercises to enhance lower limb muscle and joint stability during high-intensity exercise.

## 5. Limitations

This study, while providing valuable insights, has notable limitations. Conducted in a controlled lab setting, it does not fully replicate real-world rope skipping conditions. The focus on lower limb cushioning during forward landings overlooks other landing strategies, and the role of upper limb and trunk dynamics remains unexplored. Future research should explore the influence of landing direction and the biomechanical contributions of the upper limbs and trunk, as these factors may significantly affect joint loading and injury risk [31].

## 6. Conclusion

This SPM-based study identifies increased hip abduction, knee external rotation, and ankle internal rotation on the non-dominant side as significant predictors of lower limb injuries. Additionally, higher landing heights markedly increase vertical GRF, leading to greater joint loading and heightened injury risk. These findings emphasize the need for focused strength training of the non-dominant limb and careful regulation

of landing heights to optimize impact absorption and minimize joint stress. Future research should integrate these insights with in-depth biomechanical analyses to develop evidence-based training protocols and injury prevention strategies, ultimately improving both performance and safety in rope skipping.

**Declaration of competing interest**

The authors declare no conflicts of interest, financial or personal, that could have influenced the research and findings.

**Data availability**

Data will be made available on request.

**Acknowledgements**

The authors thank all participants for their valuable contributions and the Biomechanics Laboratory at Yanshan University for providing the experimental facilities that enabled this research.

**Appendix A. Supplementary data**

The following are the Supplementary data to this article:

[金山文档] data of paper.zip

# References

[1] Lin, Zongyu, et al. "High-fiber diet and rope-skipping benefit cardiometabolic health and modulate gut microbiota in young adults: a randomized controlled trial." Food Research International 173 (2023): 113421. https://doi.org/10.1016/j.foodres.2023.113421.

[2] Xu, D., Jiang, X., Cen, X., Baker, J. S., & Gu, Y. (2020). Single-leg landings following a

volleyball spike may increase the risk of anterior cruciate ligament injury more than landing on both-legs. Applied Sciences, 11(1), 130. https://doi.org/10.3390/app11010130.

[3] Zaheri, R. M., Majlesi, M., & Fatahi, A. (2024). The effects of fatigue on the relationship between ankle angle at initial contact and the knee and hip joints in landing: Assessing the risk of ACL injury. Gait & Posture, 113, 462-467. https://doi.org/10.1016/j.gaitpost.2024.07.299.

[4] Xu, D., Zhou, H., Wang, M., Ma, X., Gusztav, F., Chon, T. E., ... & Gu, Y. (2024). Contribution of ankle motion pattern during landing to reduce the knee-related injury risk. Computers in Biology and Medicine, 180, 108965. https://doi.org/10.1016/j.compbiomed.2024.108965.

[5] SN, Z. (2000). Contributions of lower extremity joints to energy dissipation during landings. Med Sci Sports Exerc, 32, 812-819. https://doi.org/10.1097/00005768-200004000-00014.

[6] Polk, J. D., Stumpf, R. M., & Rosengren, K. S. (2017). Limb dominance, foot orientation and functional asymmetry during walking gait. Gait & posture, 52, 140-146. https://doi.org/10.1016/j.gaitpost.2016.11.028.

[7] Gabbard, C., & Hart, S. (1996). A question of foot dominance. The Journal of general psychology, 123(4), 289-296. https://doi.org/10.1080/00221309.1996.9921281.

[8] Hart, N. H., Newton, R. U., Weber, J., Spiteri, T., Rantalainen, T., Dobbin, M., ... & Nimphius, S. (2020). Functional basis of asymmetrical lower-body skeletal morphology in professional Australian rules footballers. The Journal of Strength & Conditioning Research, 34(3), 791-799. https://doi.org/10.1519/JSC.0000000000002841.

[9] Dos’ Santos, T., Thomas, C., & Jones, P. A. (2021). Assessing interlimb asymmetries: Are we heading in the right direction?. Strength & Conditioning Journal, 43(3), 91-100. https://doi.org/10.1519/SSC.0000000000000590.

[10] Krosshaug, T., Nakamae, A., Boden, B. P., Engebretsen, L., Smith, G., Slauterbeck, J. R., ... & Bahr, R. (2007). Mechanisms of anterior cruciate ligament injury in basketball: video analysis of 39 cases. The American journal of sports medicine, 35(3), 359-367. https://doi.org/10.1177/0363546506293899.

[11] Laughlin, W. A., Weinhandl, J. T., Kernozek, T. W., Cobb, S. C., Keenan, K. G., & O'Connor, K. M. (2011). The effects of single-leg landing technique on ACL loading. Journal of biomechanics, 44(10), 1845-1851. https://doi.org/10.1016/j.jbiomech.2011.04.010.

[12] Leporace, G., Pereira, G. R., Nadal, J., & Batista, L. A. (2011). Differences in time–frequency representation of lower limbs myoelectric activity during single and double leg landing in male athletes. Journal of Electromyography and Kinesiology, 21(3), 506-511. https://doi.org/10.1016/j.jelekin.2010.12.001.

[13] Wattananon, P., Kongoun, S., Chohan, A., & Richards, J. (2023). The use of statistical parametric mapping to determine altered movement patterns in people with chronic low back pain. Journal of Biomechanics, 153, 111601. https://doi.org/10.1016/j.jbiomech.2023.111601.

[14] Pataky, T. C. (2010). Generalized n-dimensional biomechanical field analysis using statistical parametric mapping. Journal of biomechanics, 43(10), 1976-1982. https://doi.org/10.1016/j.jbiomech.2010.03.008.

[15] Pataky, T. C., Robinson, M. A., & Vanrenterghem, J. (2013). Vector field statistical analysis of kinematic and force trajectories. Journal of biomechanics, 46(14), 2394-2401. https://doi.org/10.1016/j.jbiomech.2013.07.031.

[16] Pataky, T. C., Vanrenterghem, J., & Robinson, M. A. (2015). Zero-vs. one-dimensional, parametric vs. non-parametric, and confidence interval vs. hypothesis testing procedures in

one-dimensional biomechanical trajectory analysis. Journal of biomechanics, 48(7), 1277-1285. https://doi.org/10.1016/j.jbiomech.2015.02.051.

[17] Penny, W. D., Friston, K. J., Ashburner, J. T., Kiebel, S. J., & Nichols, T. E. (Eds.). (2011). Statistical parametric mapping: the analysis of functional brain images. Elsevier. https://doi.org/10.1016/B978-0-12-372560-8.X5000-1.

[18] Castro, M. P., Pataky, T. C., Sole, G., & Vilas-Boas, J. P. (2015). Pooling sexes when assessing ground reaction forces during walking: Statistical Parametric Mapping versus traditional approach. Journal of Biomechanics, 48(10), 2162-2165. https://doi.org/10.1016/j.jbiomech.2015.05.027.

[19] Yeow, C. H., Lee, P. V. S., & Goh, J. C. H. (2009). Effect of landing height on frontal plane kinematics, kinetics and energy dissipation at lower extremity joints. Journal of biomechanics, 42(12), 1967-1973. https://doi.org/10.1016/j.jbiomech.2009.05.017.

[20] Monfort-Torres, G., García-Massó, X., Skýpala, J., Blaschová, D., & Estevan, I. (2024). Coordination and coordination variability during single-leg drop jump landing in children. Human movement science, 96, 103251. https://doi.org/10.1016/j.humov.2024.103251.

[21] de Oliveira, I., Stoelben, K. J., Tulius, E. S., de Pereira Ferreira, V., & Carpes, F. P. (2024). Strength and clinical test combinations enhance predictions of sagittal and frontal plane biomechanics in single-leg landing. Physical Therapy in Sport, 69, 1-7. https://doi.org/10.1016/j.ptsp.2024.06.008.

[22] Kristianslund, E., Krosshaug, T., & Van den Bogert, A. J. (2012). Effect of low pass filtering on joint moments from inverse dynamics: implications for injury prevention. Journal of biomechanics, 45(4), 666-671. https://doi.org/10.1016/j.jbiomech.2011.12.011.

[23] Lenz, A. L., Strobel, M. A., Anderson, A. M., Fial, A. V., MacWilliams, B. A., Krzak, J. J., & Kruger, K. M. (2021). Assignment of local coordinate systems and methods to calculate tibiotalar and subtalar kinematics: a systematic review. Journal of biomechanics, 120, 110344. https://doi.org/10.1016/j.jbiomech.2021.110344.

[24] Elvin, N. G., Elvin, A. A., Arnoczky, S. P., & Torry, M. R. (2007). The correlation of segment accelerations and impact forces with knee angle in jump landing. Journal of Applied Biomechanics, 23(3), 203-212. https://doi.org/10.1055/s-2007-965021.

[25] Chappell, J. D., Yu, B., Kirkendall, D. T., & Garrett, W. E. (2002). A comparison of knee kinetics between male and female recreational athletes in stop-jump tasks. The American journal of sports medicine, 30(2), 261-267. https://doi.org/10.1177/03635465020300021901.

[26] Wakabayashi, K., Ogasawara, I., Suzuki, Y., Nakata, K., & Nomura, T. (2022). Exploring pre-impact landing kinematics associated with increase and decrease in the anterior cruciate ligament injury risk. Journal of Biomechanics, 145, 111382. https://doi.org/10.1016/j.jbiomech.2022.111382.

[27] Ali, N., Robertson, D. G. E., & Rouhi, G. (2014). Sagittal plane body kinematics and kinetics during single-leg landing from increasing vertical heights and horizontal distances: Implications for risk of non-contact ACL injury. The Knee, 21(1), 38-46. https://doi.org/10.1016/j.knee.2012.12.003.

[28] Devita, P. A. U. L., & Skelly, W. A. (1992). Effect of landing stiffness on joint kinetics and energetics in the lower extremity. Med Sci Sports Exerc, 24(1), 108-115.

[29] Yu, B., Lin, C. F., & Garrett, W. E. (2006). Lower extremity biomechanics during the landing of a stop-jump task. Clinical biomechanics, 21(3), 297-305.

https://doi.org/10.1016/j.clinbiomech.2005.11.003.

[30] Leppänen, M., Pasanen, K., Krosshaug, T., Kannus, P., Vasankari, T., Kujala, U. M., ... & Parkkari, J. (2017). Sagittal plane hip, knee, and ankle biomechanics and the risk of anterior cruciate ligament injury: a prospective study. Orthopaedic journal of sports medicine, 5(12), 2325967117745487. https://doi.org/10.1177/2325967117745487.

[31] Shimokochi, Y., Ambegaonkar, J. P., Meyer, E. G., Lee, S. Y., & Shultz, S. J. (2013). Changing sagittal plane body position during single-leg landings influences the risk of non-contact anterior cruciate ligament injury. Knee Surgery, Sports Traumatology, Arthroscopy, 21, 888-897. https://doi.org/10.1007/s00167-012-2011-9.

 Click here to access/download;Figure;Figures.docx

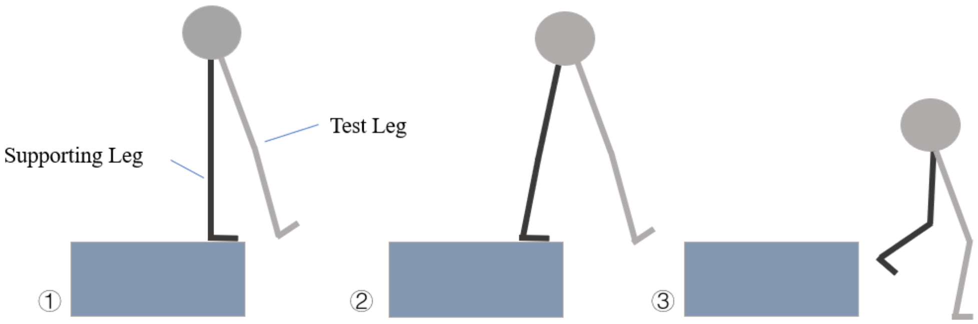


Fig.1. Single leg drop test manoeuvre

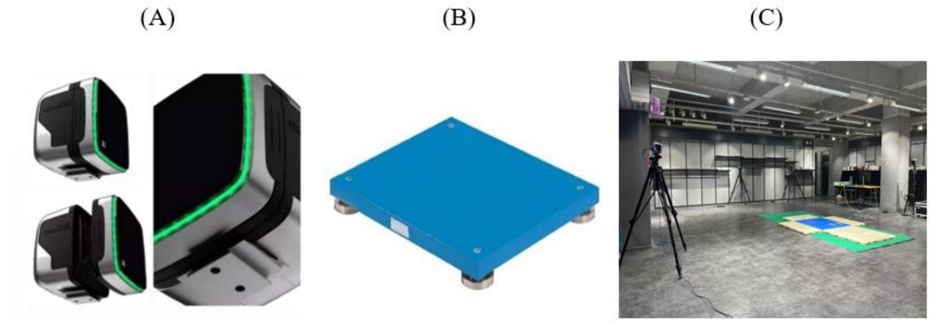


**Fig. 2.** Qualisys 3D Motion Capture System and Kistler 3D Force Stage

Note: The test apparatus included: a Qualisys 3D motion capture system to capture motion in space (A), a Kistler 3D force table to measure changes in force (B), and an experimental test site (C).

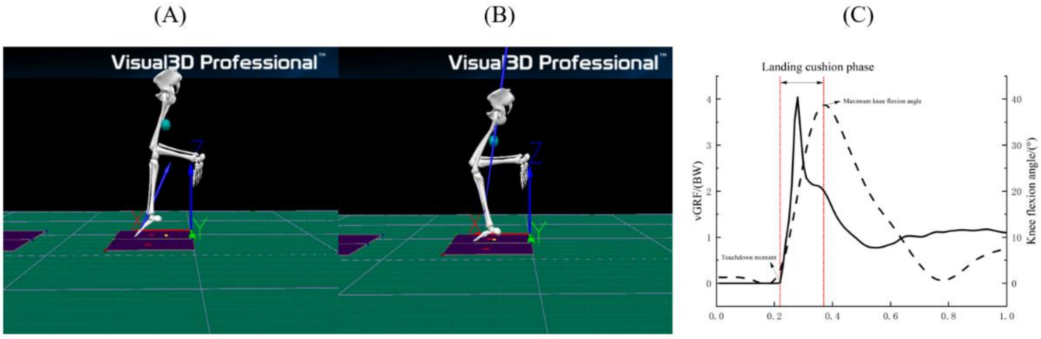


**Fig. 3.** Landed buffer phase

Note: The landing cushioning step is defined as the moment from the initial foot contact with the force platform to the maximum knee flexion angle, A: the moment from the initial foot contact with the force platform, B: the moment from the maximum knee flexion angle, C: the division of the landing cushioning phase.

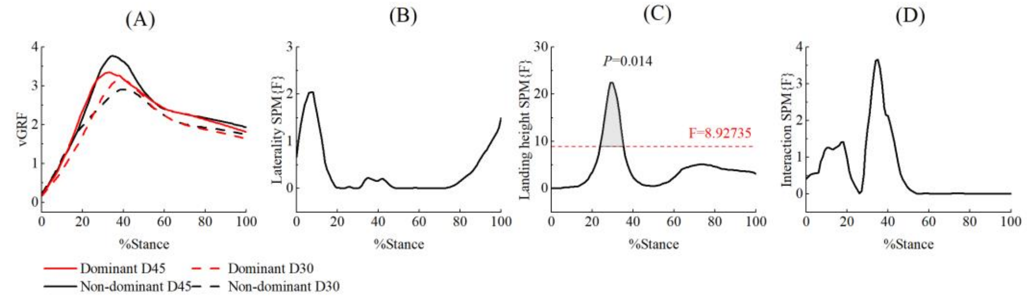


**Fig. 4.** Comparison of vertical GRF SPM{F} in the landing cushion phase for different side and drop heights. Note: Mean ensemble curves for vGRF (A) and their respective SPM{F} outputs (B, C, D) from 0 to 100% of stance phase. The dashed horizontal line on the SPM{F} outputs indicates the critical threshold boundary. The grey, shaded regions indicates the significant supra-threshold clusters representing statistically significant differences.

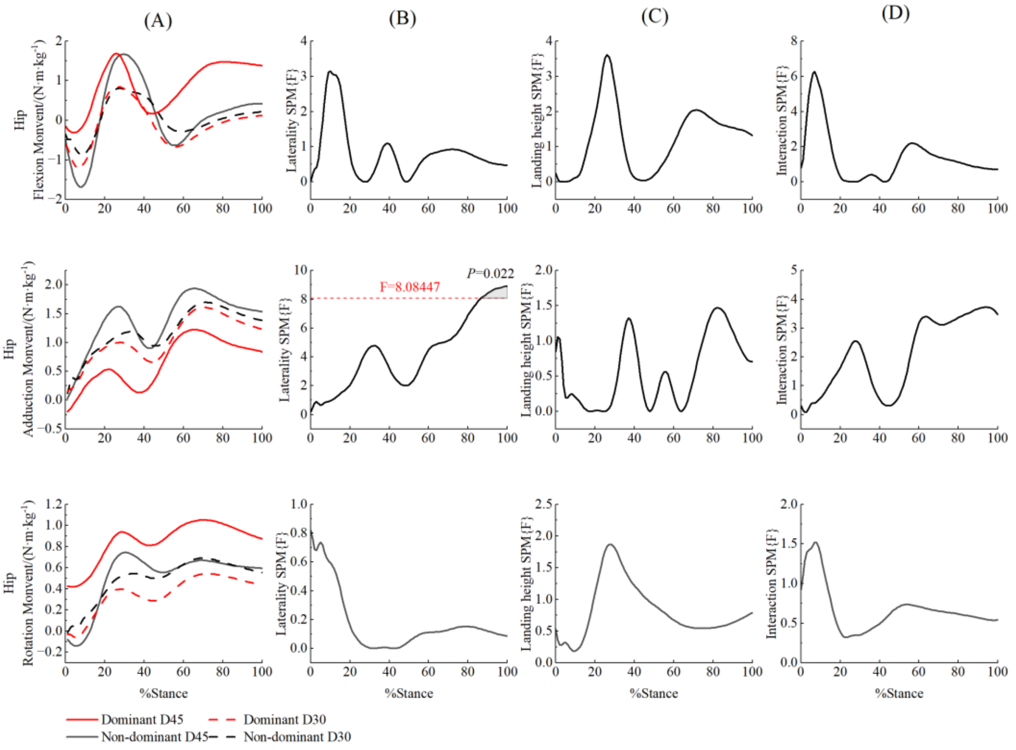


**Fig. 5.** Comparison of hip moments SPM{F} during the landing cushion phase for different side differences and drop heights.

Note: Mean ensemble curves for 3D hip joint moment (A) and their respective SPM{F} outputs (B, C, D) from 0 to 100% of stance phase. The dashed horizontal line on the SPM{F} outputs indicates the critical threshold boundary. The grey, shaded regions indicates the significant supra-threshold clusters representing statistically significant differences. Positive sagittal, frontal, and transverse plane knee joint kinetics represent internally applied knee extension/adduction/internal rotation moments.

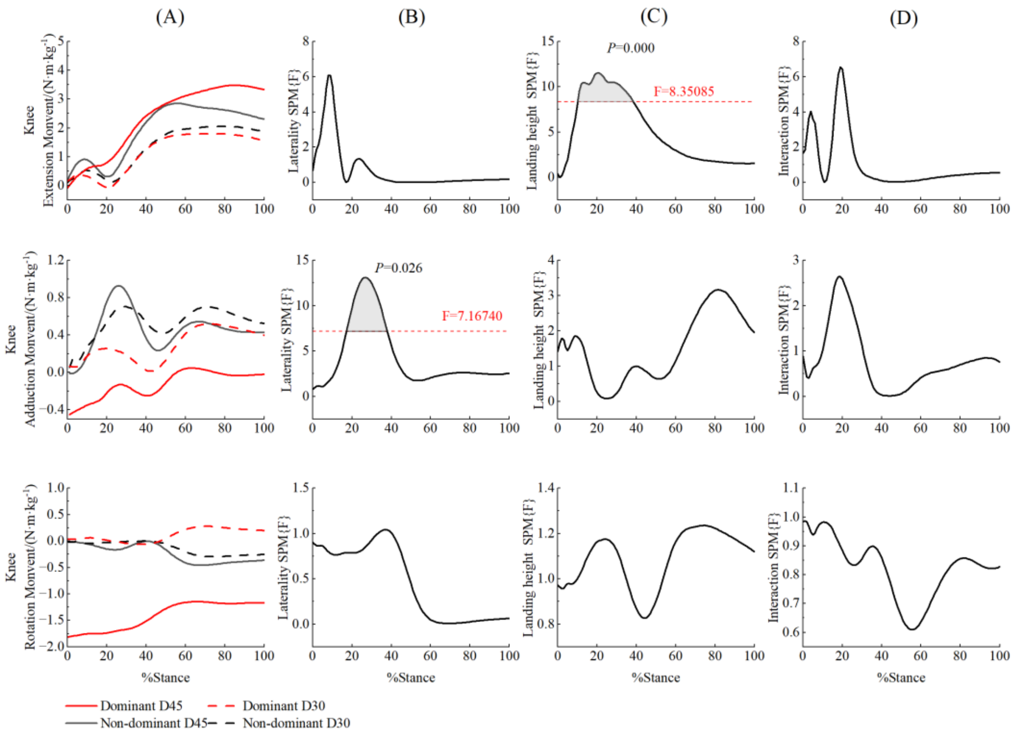


**Fig. 6.** Comparison of knee moments SPM{F} during the landing cushion phase for different side and drop heights.

Note: Mean ensemble curves for 3D knee joint moment (A) and their respective SPM{F} outputs (B, C, D) from 0 to 100% of stance phase. The dashed horizontal line on the SPM{F} outputs indicates the critical threshold boundary. The grey, shaded regions indicates the significant supra-threshold clusters representing statistically significant differences. Positive sagittal, frontal, and transverse plane knee joint kinetics represent internally applied knee extension/adduction/internal rotation moments.

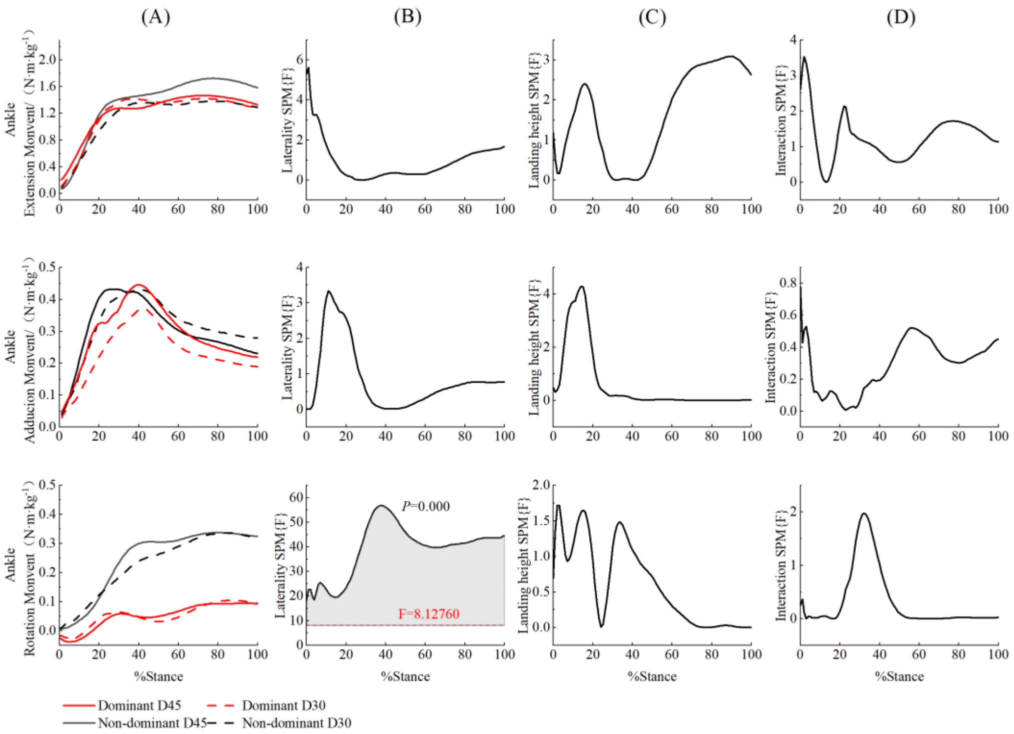


**Fig. 7.** Comparison of ankle moment SPM{F} during the landing cushioning phase for different side and drop heights.

Note: Mean ensemble curves for 3D ankle joint moment (A) and their respective SPM{F} outputs (B, C, D) from 0 to 100% of stance phase. The dashed horizontal line on the SPM{F} outputs indicates the critical threshold boundary. The grey, shaded regions indicates the significant supra-threshold clusters representing statistically significant differences. Positive sagittal, frontal, and transverse plane knee joint kinetics represent internally applied knee extension/adduction/internal rotation moments.
