# Supplementary material for: Analysis of the impact of different landing heights and lateralities on lower limb joint load based on statistical parametric mapping
Source: PLoS One. 2025 May 30;20(5):e0324656. doi: 10.1371/journal.pone.0324656 (PMC12124505; doi:10.1371/journal.pone.0324656)
Supplement: Attachment — Submitted filename: PONE-D-25-09215_R1_reviewer_reviewers comments.pdf [file pone.0324656.s004.pdf]

PLOS ONE

Analysis of the Impact of Different Landing Heights and Lateralities on Lower Limb Joint Load Based on Statistical Parametric Mapping

--Manuscript Draft--

**Manuscript Number:**	PONE-D-25-09215R1
**Article Type:**	Research Article
**Full Title:**	Analysis of the Impact of Different Landing Heights and Lateralities on Lower Limb Joint Load Based on Statistical Parametric Mapping
**Short Title:**	Landing Height and Lower Limb Joint Load
**Corresponding Author:**	Yuan Gao, PH.D<br>Yanshan University<br>Qinhuangdao, HEHebei Province CHINA
**Keywords:**	Statistical parameter mapping; Landing height; Lateralities; Lower limb joint loading
**Abstract:**	This study aimed to investigate the effects of landing height and limb laterality on lower limb joint loading using Statistical Parametric Mapping (SPM), thereby providing insights for injury prevention and optimizing performance in dynamic sports. Twenty male participants were assessed using the Qualisys 3D motion capture system and Kistler 3D force plates during landings from 30 cm and 45 cm on both dominant and non-dominant legs. No significant interaction effects between landing side and height were observed, but landing on the non-dominant leg increased the ankle joint internal rotation moment. The knee's abduction moment significantly increased during the 17%-37% landing phase, and the hip's abduction moment significantly increased during the 86%-100% phase. Higher landing height significantly increased vertical ground reaction force (GRF) during the mid-buffering phase (23%-35%). Landing on the dominant side was linked to an elevated risk of lower limb joint injuries due to increased abduction moments at the hip, external rotation moments at the knee, and internal rotation moments at the ankle. Furthermore, greater landing height correlated with higher vertical GRF, intensifying stress on the lower limbs and increasing injury risk. To reduce the risk of injury, training should focus on increasing strength and control of the non-dominant leg and controlling landing height.
**Order of Authors:**	Lian Duan, PH.D
	Yuan Gao, PH.D
	Feng Gao, PH.D
	Yang SUN, M.S
	Naiyuan Tian, M.S
**Response to Reviewers:**	Response to Reviewer #1<br><br>Thank you for your valuable suggestions, here are the specific changes we have made：<br><br>1.Errors in the text involving tenses and grammar have been corrected in accordance with your suggestions.<br><br>2.A new paragraph has been added to line 61 based on your suggestion.<br><br>3.Thank you for your valuable question regarding participant recruitment and quantity selection. In response, we have modified the original paragraph to read;<br>Study participants were recruited between 15 April 2024 and 23 April 2024 through campus bulletin board postings and social media platforms targeting university students at Yanshan University, Qinhuangdao, Hebei Province. The sample size was determined using G*Power software with the following parameters: effect size f = 0.4, α error probability = 0.05, power (1-β) = 0.8, number of groups = 1, number of measurements = 2, and correlation among repeated measures = 0.5. The minimum required sample size was calculated to be 15 participants [19]. To account for potential

data loss due to movement failure, marker displacement, or invalid data, a 30% redundancy was added, Thus, 20 male rope-skipping enthusiasts participated in this study (age: 19.75 ± 0.86 years, height: 181.24 ± 4.62 cm, body mass : 72.21± 7.66 kg, BMI: 22.06 ± 2.94 kg/m²). Data collection was completed during a 12-day experimental period following recruitment.

4.We have corrected the terminology to refer to "body mass" instead of "weight" to accurately reflect the use of kilograms (kg) as the unit of measurement.

5.In response to your question about the exclusion criteria for subjects, we have added a detailed description of the inclusion and exclusion criteria in Section Participants. which reads:
All participants were recruited based on the following inclusion criteria: (1) no congenital lower extremity deformities or acquired musculoskeletal disorders; (2) no history of lower extremity joint injuries within the past 6 months; and (3) sufficient physical ability to perform the task of stair walking. Exclusion criteria included (1) a history of lower extremity surgery; (2) use of medications affecting neuromuscular function within the past 12 months; (3) the presence of neuromuscular, psychologic, or cardiovascular disorders; and (4) the presence of acute pain symptoms during the test.

6.In response to your question about subjects familiarizing themselves with the movements and warming up. We have added content notes to the “Single Leg Landing Test” section as follows:
Prior to the formal test, participants underwent a 6-minute familiarization process, including a movement demonstration and practice trials. Additionally, a 10-minute warm-up was conducted, consisting of 5 minutes of jogging and dynamic stretching, followed by 5 minutes of light jumping and landing exercises to prepare participants for the task.

7.Regarding your question: who placed the markers? what was their level of experience? We have added a description of the relevant content in the Data collection section, as follows:
All markers were applied by the same experienced and professionally trained researcher, following standardized procedures and anatomical landmark definitions to ensure data accuracy and consistency.

8.In response to your suggestion, added references to CAST lower extremity modeling.
[23] Cappozzo, A., Catani, F., Della Croce, U., & Leardini, A. (1995). Position and orientation in space of bones during movement: anatomical frame definition and determination. Clinical biomechanics, 10(4), 171-178. https://doi.org/10.1016/0268-0033(95)91394-T.

9.Regarding your insightful question about data filtering.
In this study, a fourth-order Butterworth low-pass filter with a cutoff frequency of 12 Hz was mainly applied to smooth the trajectory data. The joint torque data were not filtered because they were derived using inverse dynamics, where the input data (including motion trajectory and filtered ground reaction force (GRF) data) were processed before calculation. Although the raw GRF data were collected using a high-precision Kistler force plate, the same 12 Hz low-pass filter was applied to the GRF data to ensure consistency with the frequency characteristics of the motion data, so the torque data itself did not need secondary filtering.

10. Regarding your question about the statement of Joint moments.
The moments mentioned in this study refer to joint moments, which are calculated by inverse dynamics methods, not internal net moments. Joint moments are calculated based on kinematic trajectories and ground reaction force (GRF) data.

11.Regarding the incorrect structure of the first sentence of the result section, I have modified it. The specific content is as follows:
Two-way ANOVA and SPM{F} analyses were performed to examine the effects of laterality (dominant vs non-dominant) and landing height (30 cm vs 45 cm) on lower extremity joint loads. The results showed that there was no significant interaction between laterality and height ($P > 0.05$). This suggests that these two factors independently affect joint loads and have no combined effect.

Powered by Editorial Manager® and ProduXion Manager® from Aries Systems Corporation

12.Added the p-values in Table 2 based on your suggestion, and added explanation for the peak moment in the table.

13.According to your suggestion, the problem to be solved and the purpose of the study are explained before the discussion begins. The specific contents is as follows:
Previous studies have primarily focused on single-limb mechanics, assuming bilateral symmetry, which limits understanding of limb asymmetries in dynamic activities. Given the high incidence of lower limb injuries, examining these differences is crucial. This study aimed to investigate the effects of landing height and limb lateralities on joint loading, addressing this gap and providing insights for injury prevention and performance optimization.

14.Regarding the description of the positive correlation in the cited Leppänen article, it has been revised in the text as follows:
This is consistent with Leppänen et al. [32], which reported a significant positive correlation between knee flexion moments and the risk of ACL injury. Specifically, for every 10 N·m increase in knee flexion moment, the risk of ACL injury increased by 21% (HR = 1.21, 95% CI: 1.04–1.40, p = 0.01) [2, 20].

Because Leppänen et al. reported hazard ratios (HR) rather than correlation coefficients (r). Due to methodological differences, the assessment of ACL injury risk is typically conducted using survival analysis and regression models; therefore, r values were not provided in their study.

Response to Reviewer #2

1.Reviewer #2: Suggestion: In the abstract, you use “knee's external abduction moment” (row 30), and “hip's external abduction moment” (row 31). There is no concept like “external abduction”. If you say that the moment is external, so how should we understand the “ankle joint internal rotation moment” (row 29)? Like internal moment or internal rotation in the ankle? Furthermore, you never mention them again in the text.

Author: Thank you for pointing out the problem of inconsistent and ambiguous terminology describing joint moments in the abstract. We agree that phrases such as “external abduction moment” are imprecise and may cause confusion. To clarify, we have revised the wording as follows: “The knee's abduction moment significantly increased during the 17%–37% landing phase, and the hip's abduction moment significantly increased during the 86%–100% phase.”

2.Reviewer #2: The purpose of the study is not clearly stated.

Author: Thank you for your helpful suggestions on clarifying the purpose of the study. In response, we have revised the opening sentence of the Abstract to explicitly state the aim of the study. In addition, we have updated the final paragraph of the Introduction to clearly articulate the specific objectives, research focus, and intended contributions of the work.

3.Reviewer #2: Your introduction section focuses entirely on the rope jumping process, while the study only includes landing from different heights, without a preceding flight phase. Indeed this investigation could relate to many other “landing” activities (for example all kinds of jumps). A revision in this section is needed. You perform the experimental investigation with rope-skipping enthusiasts, not professionals. How often do these enthusiasts practice rope-skipping, and do they practice other sports, leading to different leg strengths?

Author: Thank you for your insightful comments regarding the focus of the introduction and the sample population studied. Regarding the focus on rope skipping, we acknowledge that the introduction may have emphasized this specific activity. However, the primary goal of the study was to explore landing mechanics, which are not limited to rope skipping but extend to various dynamic landing activities such as

jumps from different heights. The focus on rope skipping enthusiasts was driven by the practical need to assess biomechanics in a commonly practiced activity where landing injuries are prevalent, thus providing relevant data for injury prevention and performance optimization in this context.
In terms of the sample population, we understand the importance of leg strength and the potential impact of training frequency on biomechanics. However, the participants in this study were specifically selected to represent rope skipping enthusiasts with regular training, and we did not explore additional factors such as cross-training or other sports. While the potential variability in leg strength could be considered in future research, the current study's aim was to focus on the biomechanical effects of landing height and limb lateralities, which are independent of the participants' leg strength or training history. Future studies could expand this research by incorporating athletes with different training backgrounds or exploring variations in landing techniques across a broader range of activities.

Reviewer #2: During standard rope-skipping man jump approximately 15-30 sm in height. How did you determine your 30 and 45 sm?

Author: Thank you for your valuable comments regarding the selection of 30 cm and 45 cm as landing heights. In standard rope skipping, the vertical displacement during flight is relatively small (typically 15–30 cm), as noted. However, the heights of 30 cm and 45 cm were intentionally selected to simulate increased landing intensity conditions, which are commonly encountered in freestyle rope skipping or other jumping-related activities where athletes perform complex aerial movements before landing.
These controlled drop heights are widely used in biomechanical research to safely reproduce variable landing loads and assess their effects on joint kinetics and kinematics under standardized conditions. The use of 30 cm and 45 cm also aligns with prior studies analyzing single-leg or drop landings to provoke meaningful biomechanical responses, particularly in joint loading and ground reaction forces. Therefore, while these heights may exceed typical skipping jump heights, they provide a practical and validated model for investigating the effects of increased landing demand, which is relevant not only to rope skipping but to various athletic movements involving unilateral landings.

4.Reviewer #2: Please add a citation for “CAST lower limb model” and explain the abbreviation.
Author: Thank you for pointing out this important problemand we have made the following changes.
During trials, participants wore study-specific tights, and after warming up, 20 infrared reflective markers were placed on anatomical landmarks according to the CAST (Calibrated Anatomical Systems Technique) lower limb model, which employs static calibration and marker tracking to establish joint coordinate systems while minimizing soft tissue artifacts [23].
[23]Cappozzo, A., Catani, F., Della Croce, U., & Leardini, A. (1995). Position and orientation in space of bones during movement: anatomical frame definition and determination. Clinical biomechanics, 10(4), 171-178. https://doi.org/10.1016/0268-0033(95)91394-T.

5.Reviewer #2: In addition to photos of the equipment, you can also add one or some participants during the experiment.

Author: Thank you for your suggestion, to which we added images of the participants.

Fig. 3. Participants performing the single-leg landing task from two different heights (30 cm and 45 cm).
Note: Participants stood on the jumping box with one leg lifted, then leaned forward and landed on the test leg while keeping their hands on their hips. The two heights correspond to the experimental conditions used for data collection.

6.Reviewer #2: “This study analyzes biomechanical indicators …” (row 132). Which are they?
Author: We appreciate your attention to detail. The biomechanical indicators analyzed in this study include vertical ground reaction force (vGRF) and three-dimensional joint

moments of the hip, knee, and ankle during the landing cushioning phase. Specifically, the joint moments include:
Hip: flexion/extension, abduction/adduction, internal/external rotation
Knee: flexion/extension, varus/valgus, internal/external rotation
Ankle: plantarflexion/dorsiflexion, inversion/eversion, internal/external rotation
These indicators are described in detail in Section Statistical Analysis and summarized throughout the Results section (Tables 1–2 and Figures 4–7).

7.Reviewer #2: “The hip joint stabilizes the coronal plane by increasing abduction moments…” (row 229). This sentence structure is not correct. Hip joints do not stabilize any plane. Abduction movement is performed in that plane.

Author: Thank you for your insightful comments! We agree that the hip joint itself does not stabilize any anatomical plane. Rather, we intended to express that hip abduction torque, which occurs in the frontal plane, contributes to dynamic postural control during landing. We have revised the sentence accordingly to avoid misinterpretation.
During landing, increased hip abduction moment in the frontal plane contributes to maintaining postural stability through coordinated muscular control and compensatory movement strategies.

8.Reviewer #2: “Excessive hip abduction can shift the center of pressure outward, leading to compensatory changes in adjacent joints, increasing knee valgus and ACL injury risk ” (row 232). During drop landing, you do not observe “excessive hip abduction” which is over 50 degrees in the joint, but only increased abduction moment.

Author: Thank you for pointing this out. We agree that “excessive hip abduction” may be misleading in the context of our findings, as our study did not assess hip abduction angles but rather observed increased hip abduction moments on the non-dominant side during landing. We have revised the sentence to accurately reflect this and avoid confusion between joint angle and joint moment.
The revised paragraph reads: Increased hip abduction moment may shift the center of pressure laterally, leading to compensatory changes in adjacent joints and potentially increasing the risk of knee valgus and ACL injury.

9.Reviewer #2: Rows 251-255. I recommend you 1) move it to the conclusion section; 2) do not be so confident in speculating the results with the need for “strength training ”. Joint stability depends on bone shape congruency, capsule and ligament elasticity, muscle strength, neuromuscular control, fatigue. Better find some previous literature sources, and think in the field of coordination, and motor control.

Author: Thank you for the valuable feedback. We agree that joint stability is a multifactorial phenomenon influenced by not only muscle strength but also ligament properties, joint congruency, neuromuscular control, and fatigue. In response, the statement has been moved to the conclusion section as recommended.

10.Reviewer #2: Some citations are missing from the text like 20, 22, 23…

Thank you for pointing out the problem regarding missing citations. Upon careful review, we would like to clarify that reference 20 is cited in Section Participants of the manuscript. Reference 22 is included in Section Single-leg landing test. References 23 and 24 are cited in the Data collection and Data processing sections, respectively. Some of these references also appear later as cross-references to support relevant discussions. We have carefully checked the entire manuscript to ensure that all citations are accurate and consistently presented.

Thanks again for your valuable suggestions.

Response to journal

Dear Editors,

Thank you very much for your helpful suggestions and for the opportunity to revise our

	manuscript. We have carefully addressed all the requested points based on the recommendations of the journal and the two reviewers and we sincerely hope that our revised manuscript will be considered for publication in PLOS ONE. We truly value the academic quality and open-access mission of your journal, and we are very eager to share our findings with the broader scientific community through your platform.<br><br>Please find below a summary of the revisions made:<br><br>Manuscript Formatting: The layout of the manuscript has been modified in accordance with the PLOS ONE submission style template to ensure compliance with the journal's formatting guidelines.<br><br>ORCID Updates: The ORCID iDs of all corresponding authors have been updated in the submission system as requested.<br><br>Data Availability: We confirm that the manuscript includes all raw data necessary to replicate the results presented in our study.<br><br>Author Information: The author list has been updated. The first author now includes two institutional affiliations, and we have clearly designated three corresponding authors.<br><br>Figures Submission: All figures have been removed from the main manuscript and are now uploaded separately as individual high-resolution files, following PLOS ONE's submission requirements.<br><br>We thank you again for your time and consideration. We look forward to the possibility of publishing in PLOS ONE and sharing our work with your global readership.<br><br>Thanks again for your valuable suggestions.<br><br>Sincerely,<br>Yuan Gao<br>On behalf of all co-authors
**Additional Information:**	
**Question**	**Response**
**Financial Disclosure**<br><br>Enter a financial disclosure statement that describes the sources of funding for the work included in this submission. Review the submission guidelines for detailed requirements. View published research articles from *PLOS ONE* for specific examples.<br><br>This statement is required for submission and **will appear in the published article** if the submission is accepted. Please make sure it is accurate.	The author(s) received no specific funding for this work.

**Funded studies**<br>Enter a statement with the following details:<br>• Initials of the authors who received each award<br>• Grant numbers awarded to each author<br>• The full name of each funder<br>• URL of each funder website<br>• Did the sponsors or funders play any role in the study design, data collection and analysis, decision to publish, or preparation of the manuscript?<br><br>Did you receive funding for this work?	
**Competing Interests**<br><br>Use the instructions below to enter a competing interest statement for this submission. On behalf of all authors, disclose any competing interests that could be perceived to bias this work—acknowledging all financial support and any other relevant financial or non-financial competing interests.<br><br>This statement is required for submission and **will appear in the published article** if the submission is accepted. Please make sure it is accurate and that any funding sources listed in your Funding Information later in the submission form are also declared in your Financial Disclosure statement.<br><br>View published research articles from *PLOS ONE* for specific examples.	The authors have declared that no competing interests exist

Powered by Editorial Manager® and ProduXion Manager® from Aries Systems Corporation

**NO authors have competing interests**<br><br>Enter: *The authors have declared that no competing interests exist.*<br><br>**Authors with competing interests**<br><br>Enter competing interest details beginning with this statement:<br><br>*I have read the journal's policy and the authors of this manuscript have the following competing interests: [insert competing interests here]*<br><br>* typeset	
**Ethics Statement**<br><br>Enter an ethics statement for this submission. This statement is required if the study involved:<br><br>• Human participants<br>• Human specimens or tissue<br>• Vertebrate animals or cephalopods<br>• Vertebrate embryos or tissues<br>• Field research<br><br>Write "N/A" if the submission does not require an ethics statement.<br><br>General guidance is provided below. Consult the submission guidelines for detailed instructions. **Make sure that all information entered here is included in the Methods section of the manuscript.**	Human participants<br>Approving Authority: Ethics Committee of Qinhuangdao First Hospital<br>Approval Number: 2024Y006<br>Form of approval: Written

Powered by Editorial Manager® and ProduXion Manager® from Aries Systems Corporation

**Format for specific study types**<br><br>**Human Subject Research (involving human participants and/or tissue)**<br>• Give the name of the institutional review board or ethics committee that approved the study<br>• Include the approval number and/or a statement indicating approval of this research<br>• Indicate the form of consent obtained (written/oral) or the reason that consent was not obtained (e.g. the data were analyzed anonymously)<br><br>**Animal Research (involving vertebrate animals, embryos or tissues)**<br>• Provide the name of the Institutional Animal Care and Use Committee (IACUC) or other relevant ethics board that reviewed the study protocol, and indicate whether they approved this research or granted a formal waiver of ethical approval<br>• Include an approval number if one was obtained<br>• If the study involved *non-human primates*, add *additional details* about animal welfare and steps taken to ameliorate suffering<br>• If anesthesia, euthanasia, or any kind of animal sacrifice is part of the study, include briefly which substances and/or methods were applied<br><br>**Field Research**<br><br>Include the following details if this study involves the collection of plant, animal, or other materials from a natural setting:<br>• Field permit number<br>• Name of the institution or relevant body that granted permission	
**Data Availability**<br><br>Authors are required to make all data underlying the findings described fully available, without restriction, and from the time of publication. PLOS allows rare exceptions to address legal and ethical concerns. See the PLOS Data Policy and FAQ for detailed information.	Yes - all data are fully available without restriction

A Data Availability Statement describing where the data can be found is required at submission. Your answers to this question constitute the Data Availability Statement and **will be published in the article**, if accepted.<br><br>**Important:** Stating 'data available on request from the author' is not sufficient. If your data are only available upon request, select 'No' for the first question and explain your exceptional situation in the text box.<br><br>Do the authors confirm that all data underlying the findings described in their manuscript are fully available without restriction?	
**Describe where the data may be found in full sentences. If you are copying our sample text, replace any instances of XXX with the appropriate details.**<br><br>• If the data are **held or will be held in a public repository**, include URLs, accession numbers or DOIs. If this information will only be available after acceptance, indicate this by ticking the box below. For example: *All XXX files are available from the XXX database (accession number(s) XXX, XXX.).*<br>• If the data are all contained **within the manuscript and/or Supporting Information files**, enter the following: *All relevant data are within the manuscript and its Supporting Information files.*<br>• If neither of these applies but you are able to provide **details of access elsewhere**, with or without limitations, please do so. For example:<br><br>*Data cannot be shared publicly because of [XXX]. Data are available from the XXX Institutional Data Access / Ethics Committee (contact via XXX) for researchers who meet the criteria for access to confidential data.*<br><br>*The data underlying the results presented in the study are available from (include the name of the third party*	All relevant data are within the manuscript and its Supporting Information files.

Powered by Editorial Manager® and ProduXion Manager® from Aries Systems Corporation

and contact information or URL).<br>• This text is appropriate if the data are owned by a third party and authors do not have permission to share the data.<br><br>* typeset	
Additional data availability information:	Tick here if your circumstances are not covered by the questions above and you need the journal's help to make your data available.; Tick here if the URLs/accession numbers/DOIs will be available only after acceptance of the manuscript for publication so that we can ensure their inclusion before publication.

Powered by Editorial Manager® and ProduXion Manager® from Aries Systems Corporation

Click here to access/download;Manuscript;Manuscript .docx

Analysis of the Impact of Different Landing Heights and Lateralities on Lower Limb Joint Load Based on Statistical Parametric Mapping

Lian Duan[1¶], Yuan GAO[*¶], Feng GAO[*¶], Yang SUN[*&], Naiyuan Tian[2&]

[1] China Volleyball College, Beijing Sport University, Beijing, China.

[1] School of Physical Education, Yanshan University, Qinhuangdao, Hebei, China.

[*] School of Physical Education, Yanshan University, Qinhuangdao, Hebei, China.

[*] China Volleyball College, Beijing Sport University, Beijing, China.

[*] School of Physical Education, Yanshan University, Qinhuangdao, Hebei, China.

[2] School of Physical Education, Yanshan University, Qinhuangdao, Hebei, China.

* Corresponding author

Email: gaoyuan197812@126.com (YG)

Email: gaofeng202502@126.com (FG)

Email: syang0217@126.com (YS)

¶ These authors contributed equally to this work.

& These authors also contributed equally to this work.

Abstract

This study aimed to investigate the effects of landing height and limb laterality on lower limb joint loading using Statistical Parametric Mapping (SPM), thereby providing insights for injury prevention and optimizing performance in dynamic sports. Twenty male participants were assessed using the Qualisys 3D motion capture system and Kistler 3D force plates during landings from 30 cm and 45 cm on both dominant and non-dominant legs. No significant interaction effects between landing side and height were observed, but landing on the non-dominant leg increased the ankle joint internal rotation moment. The knee's abduction moment significantly increased during the 17%-37% landing phase, and the hip's abduction moment significantly increased during the 86%-100% phase. Higher landing height significantly increased vertical ground reaction force (GRF) during the mid-buffering phase (23%-35%). Landing on the dominant side was linked to an elevated risk of lower limb joint injuries due to increased abduction moments at the hip, external rotation moments at the knee, and internal rotation moments at the ankle. Furthermore, greater landing height correlated with higher vertical GRF, intensifying stress on the lower limbs and increasing injury risk. To reduce the risk of injury, training should focus on increasing strength and control of the non-dominant leg and controlling landing height.

Keywords: Statistical parameter mapping; Landing height; Lateralities; Lower limb joint loading

Introduction

In recent years, rope skipping has emerged as a widely adopted exercise modality, owing to its low cost, ease of learning, and cardiovascular benefits. Its effectiveness in improving both aerobic fitness and coordination has made it a favored exercise among fitness enthusiasts and recreational athletes alike [1]. Freestyle rope skipping, known for its diverse techniques and visually engaging performances, has gained widespread popularity. However, the complexity of these movements elevates the risk of injury, particularly during the landing phase. Recent studies highlight a notably high incidence of lower limb injuries (87.5%), with knee and ankle joints being most frequently affected, comprising approximately 80% of all injury cases [2-4]. Despite the human body's inherent structural symmetry, functional asymmetry, or laterality, is well-documented in lower limb biomechanics [5-6]. Excessive lateral laterality has been identified as a potential risk factor for non-contact joint injuries [7-8]. However, most rope skipping research assumes limb symmetry, primarily analyzing data from a single limb to assess landing-related injury risks [9-12]. Consequently, there is limited understanding of the biomechanical differences between dominant and non-dominant limbs during single-leg landing in rope skipping, highlighting a critical gap in the literature.

Statistical Parametric Mapping (SPM), based on random field theory, utilizes topological analysis to examine dynamic changes and interrelationships in time series data, enhancing the robustness of biomechanical conclusions, as indicated by

Wattananon et al. [13] and Pataky et al. [14-16]. While traditional zero-dimensional statistical methods can identify feature differences, they often fail to capture dynamic patterns in time series data, as highlighted by previous studies [16-18]. To address these limitations, this study aimed to systematically analyze the effects of landing height and limb lateralities on lower limb joint loading using Statistical Parametric Mapping (SPM). Specifically, the study focused on biomechanical differences between the dominant and non-dominant limbs during landings from different heights and their subsequent impact on joint loading. By addressing this gap in the literature, the study aims to provide scientific evidence for injury prevention in dynamic activities such as rope skipping and to optimize training strategies.

Materials and methods

Participants

Study participants were recruited between 15 April 2024 and 23 April 2024 through campus bulletin board postings and social media platforms targeting university students at Yanshan University, Qinhuangdao, Hebei Province. The sample size was determined using G*Power software with the following parameters: effect size f = 0.4, α error probability = 0.05, power (1-β) = 0.8, number of groups = 1, number of measurements = 2, and correlation among repeated measures = 0.5. The minimum

required sample size was calculated to be 15 participants [19]. To account for potential data loss due to movement failure, marker displacement, or invalid data, a 30% redundancy was added, Thus, 20 male rope-skipping enthusiasts participated in this study (age: 19.75 ± 0.86 years, height: 181.24 ± 4.62 cm, body mass：72.21± 7.66 kg, BMI: 22.06 ± 2.94 kg/m²). Data collection was completed during a 12-day experimental period following recruitment.

All participants were recruited based on the following inclusion criteria: (1) no congenital lower extremity deformities or acquired musculoskeletal disorders; (2) no history of lower extremity joint injuries within the past 6 months; and (3) sufficient physical ability to perform the task of stair walking. Exclusion criteria included (1) a history of lower extremity surgery; (2) use of medications affecting neuromuscular function within the past 12 months; (3) the presence of neuromuscular, psychologic, or cardiovascular disorders; and (4) the presence of acute pain symptoms during the test. All procedures were in accordance with the Declaration of Helsinki. Participants were fully informed of the purpose and procedures of the study and written informed consent was obtained. The study protocol was approved by the Ethics Committee of Qinhuangdao First Hospital.

The determination of the dominant side was conducted through a kicking test, where participants were asked to kick with both feet. The foot that achieved the greater kicking distance was identified as the dominant side [7], [20].

Single-leg landing test

Participants were instructed to place their hands on their hips and stand 5 cm from the force platform, with jumping boxes positioned at heights of 30 cm and 45 cm. Upon receiving a "start" command, participants lifted their test leg and held it in the air. They then leaned forward and descended by inertia, landing on the test foot to maintain posture and stability [2], [21]. Participants were required to focus visually on a target displayed on a blank wall in front of them throughout the test. Prior to the formal test, participants underwent a 6-minute familiarization process, including a movement demonstration and practice trials. Additionally, a 10-minute warm-up was conducted, consisting of 5 minutes of jogging and dynamic stretching, followed by 5 minutes of light jumping and landing exercises to prepare participants for the task. Trials were repeated if participants touched the ground with the non-test leg or used their arms for balance (Fig. 1 and 2) [22].

Data collection

During trials, participants wore study-specific tights, and after warming up, 20 infrared reflective markers were placed on anatomical landmarks according to the CAST (Calibrated Anatomical Systems Technique) lower limb model, which employs static calibration and marker tracking to establish joint coordinate systems while minimizing soft tissue artifacts [23]. All markers were applied by the same

experienced and professionally trained researcher, following standardized procedures and anatomical landmark definitions to ensure data accuracy and consistency. Following the collection of static model data, eight markers were removed from the medial and lateral sides of the knee and ankle joints. Participants performed at least three complete movements during trials, and kinematic and kinetic data were synchronously captured using the Qualisys 3D motion capture system (8 cameras, 200 Hz) and a Kistler 3D force platform (2000 Hz) during landing (Fig. 3).

Data processing

In this study, the landing cushion phase (the moment of contact of the test leg on the ground up to the maximum angle of knee flexion of the supporting leg) of the drop jump movement was recorded. The raw data were pre-processed using the QTM software supplied with the Qualisys system, and hip, thigh, calf, and foot coordinate systems were established based on the CAST lower limb model, using infrared reflective markers to create a simulation of the subject's lower limb. Kinematic data were subsequently imported into Visual 3D software for further processing. A fourth-order Butterworth low-pass filter with a cutoff frequency of 12 Hz was applied to smooth the data, as described by Kristianslund et al. [24], Lenz et al. [25] and Monfort-Torres et al. [21]. Joint moments for ankle plantarflexion, knee flexion, and hip flexion (N•m•kg) were defined as positive (+) values, while ankle dorsiflexion, knee extension, and hip extension moments were defined as negative (−) values.

Ground reaction forces (N) and joint moments (N•m•kg) Lenz et al. [25] were calculated through inverse dynamics (Fig. 4).

Statistical analysis

This study analyzes biomechanical indicators related to lower limb joint loads during the landing cushioning phase, defined as the period from initial ground contact by the supporting leg to maximum knee flexion. Joint moments were normalized to 101 data points across the landing phase for precise temporal resolution. Mean value curves were generated to compare dynamic variations in joint load related parameters. A two-way ANOVA was employed to examines the effects of side and landing height on peak vertical GRF and 3D joint moments during the cushioning phase, with normality and homogeneity of variance verified. SPM{F} analysis assessed side and landing height effects on vertical GRF and 3D joint moment time series. Statistical significance was set at $p < 0.05$. Analyses were performed with SPSS 23.0 and Origin 2022.

Results

Two-way ANOVA and SPM{F} analyses were performed to examine the effects of laterality (dominant vs non-dominant) and landing height (30 cm vs 45 cm) on lower extremity joint loads. The results showed that there was no significant interaction between laterality and height ($p > 0.05$). This suggests that these two factors

independently affect joint loads and have no combined effect.

Ground reaction forces in the landing cushioning phase

The two-way ANOVA results indicated that the peak vertical GRF during the landing cushioning phase was significantly greater for the 45 cm drop height (4.32 BW) compared to the 30 cm drop height (3.48 BW), independent of the side factor, with statistical significance denoted by $p < 0.001$ and F = 30.914 (Table 1).

Table 1. Comparison of vertical GRF peaks during the landing cushion phase for different side groups and drop heights（BW）

	**D30**	**D45**
**Non-dominant**	3.47±0.48$^{\#}$	4.31±0.64
**Dominant**	3.49±0.58	4.34±0.67

Note: Mean (Standard difference), *:There was a significant difference between D30 and D45.

The SPM{F} analysis revealed that during the 23% to 35% of the landing cushioning phase, the vertical GRF for the 45 cm drop height was significantly greater than that for the 30 cm drop height, regardless of the side factor, with $p = 0.004$ and F = 8.927 (Fig. 5).

Lower limb kinetic characteristics during the landing cushioning phase

The results of the two-way ANOVA indicate that during the landing cushioning phase,

the peak dorsiflexion moment of the ankle joint (0.99 N•m•kg^{-1} vs. 1.64 N•m•kg^{-1}, p = 0.01, F = 6.901), the peak internal rotation moment of the ankle joint (0.11 N•m•kg^{-1} vs. 0.37 N•m•kg^{-1}, $p < 0.001$, F = 47.348), the peak abduction moment of the knee joint (0.36 N•m•kg^{-1} vs. 0.88 N•m•kg^{-1}, p = 0.006, F = 7.834), and the peak external rotation moment of the knee joint (0.19 N•m•kg^{-1} vs. -0.41 N•m•kg^{-1}, $p <$ 0.001, F = 58.042), were significantly greater on the non-dominant side compared to the dominant side. In addition, irrespective of the side factor, the peak flexion moment of the knee joint when landing from a 45 cm height was significantly greater (2.66 N•m•kg^{-1} vs. 2.03 N•m•kg^{-1}, p = 0.003, F = 9.132) than when landing from a 30 cm height (Table 2).

Table 2. Comparison of the peak moments of the lower limb joints during the landing cushioning phase by side and height of fall.

**Peak moment (N·m·kg^{-1})**	**Dominant**		**Non-dominant**		**Interaction effect *p* value**	**Main effect of laterality *p* value**	**Main Effect of Height *p* value**
	**D30**	**D45**	**D30**	**D45**			
**Ankle dorsiflexion**	0.95±1.39*	1.04±1.51	1.65±0.52	1.62±0.49	0.797	0.010	0.916
**Ankle valgus**	0.46±0.36	0.60±0.45	0.49±0.41	0.52±0.34	0.538	0.849	0.337
**Ankle internal rotation**	0.11±0.15*	0.11±0.18	0.36±0.17	0.38±0.17	0.862	0.000	0.878
**Knee flexion**	1.90±1.12#	2.42±1.15	2.16±0.65	2.89±0.72	0.598	0.077	0.003
**Knee valgus**	0.20±0.71*	0.23±0.98	0.90±0.56	0.85±0.96	0.549	0.006	0.391
**Knee internal rotation**	0.22±0.33*	0.17±0.47	-0.30±0.22	-0.51±0.35	0.327	0.000	0.107
**Hip flexion**	0.52±2.21	0.74±3.17	0.57±1.72	2.05±2.14	0.239	0.204	0.117

**Hip valgus**	1.79±1.47	1.80±1.44	1.93±0.49	2.13±0.47	0.707	0.345	0.672
**Hip internal rotation**	0.61±0.31	0.64±0.57	0.78±0.30	0.83±0.46	0.968	0.065	0.658

Note: Joint Moment (Peak Joint Moment), Mean (Standard difference), *: Significant difference between dominant and non-dominant side, #: There was a significant difference between D30 and D45.

The SPM{F} analysis indicated that during the 86% to 100% of the landing cushioning phase, the peak hip abduction moment was significantly greater on the non-dominant side compared to the dominant side (($F = 8.084$, $p = 0.022$), irrespective of the height factor (Fig. 6). Similarly, during the 17% to 37% of the landing cushioning phase, the peak abduction moment of the knee joint was significantly greater on the non-dominant side (($F = 7.167$, $p = 0.026$), independent of height factor (Fig. 7). Throughout the entire landing cushioning phase, the ankle internal rotation moment on the non-dominant side was significantly greater than on the dominant side ($F = 8.127$, $p < 0.001$) (Fig. 8). In addition, during the 10% to 38% of the landing cushioning phase, the knee joint flexion moment was significantly greater for a 45 cm landing ($F = 8.350$, $p < 0.001$) than for a 30 cm landing, regardless of the side factor (Fig. 7).

Discussion and analysis

Previous studies have primarily focused on single-limb mechanics, assuming bilateral symmetry, which limits understanding of limb asymmetries in dynamic activities. Given the high incidence of lower limb injuries, examining these differences is crucial.

This study aimed to investigate the effects of landing height and limb lateralities on joint loading, addressing this gap and providing insights for injury prevention and performance optimization.

Comparative analysis of side differences during the landing cushioning phase

Two-way ANOVA showed no significant difference in peak 3D hip moments regarding side or height, but SPM{F} revealed higher hip abduction moments on the non-dominant side, reflecting the need for stability during maximum knee flexion and impact absorption [20]. During landing, increased hip abduction moment in the frontal plane contributes to maintaining postural stability through coordinated muscular control and compensatory movement strategies [20, 22]. During the landing cushioning phase, the non-dominant leg exhibits a more pronounced increase in abduction moment to compensate for uneven strength and stabilize overall posture. Increased hip abduction moment may shift the center of pressure laterally, leading to compensatory changes in adjacent joints and potentially increasing the risk of knee valgus and ACL injury [10], [26-28]. The larger hip abduction moment in the non-dominant leg during the landing cushioning phase may impact the knee joint within the kinetic chain. Additionally, while SPM{F} analysis showed a significant increase in the non-dominant leg's hip abduction moment, no significant difference in external rotation moment was observed, likely due to the conservative significance

threshold used in zero-dimensional statistical analyses to prevent Type I errors [18]. Previous research has shown that zero-dimensional models have limitations in deriving probabilistic inferences from one-dimensional dynamic data [15-17]. Although one-dimensional analysis may miss variations in knee joint external rotation, zero-dimensional models might still reveal significant differences. The SPM{F} results show that the non-dominant ankle exhibited a higher internal rotation moment during landing. This is likely due to the ankle's complex anatomy, which is inherently more prone to deformation upon impact. To compensate, the body increased the internal rotation moment for stability. Furthermore, the non-dominant side, with relatively weaker muscle strength and less precise neural control, may require greater plantarflexion and internal rotation to maintain landing stability [9].

Comparative analysis of different heights during the landing cushioning phase

Both two-way ANOVA and SPM{F} analyses confirmed that a 45 cm jump height generated significantly higher vertical GRF than a 30 cm jump. This aligns with physical principles: greater landing height increases vertical velocity with constant mass, requiring a higher impulse to counter momentum change. Due to the body's limited impact absorption capacity, excess force translates into higher GRF, amplifying landing impact. This finding supports Ali et al. [29], revealing a significant increase in knee flexion moment during landing from 45 cm compared to

30 cm. Previous studies have established that the knee joint, as the primary weight-bearing and energy-absorbing joint, plays a crucial role in impact force absorption during the cushioning phase [2], [30]. Accordingly, our study showed that higher landing heights resulted in increased impact forces, placing greater load on the knee for absorption and cushioning [31]. This is consistent with Leppänen et al. [32], which reported a significant positive correlation between knee flexion moments and the risk of ACL injury. Specifically, for every 10 N·m increase in knee flexion moment, the risk of ACL injury increased by 21% (HR = 1.21, 95% CI: 1.04–1.40, $p = 0.01$) [2], [20]. To reduce injury risk, rope jumping participants should control landing heights and gradually increase them during training. Complementing this with strength and coordination exercises to enhance lower limb muscle and joint stability during high-intensity exercise.

Limitations

This study, while providing valuable insights, has notable limitations. Conducted in a controlled lab setting, it does not fully replicate real-world rope skipping conditions. The focus on lower limb cushioning during forward landings overlooks other landing strategies, and the role of upper limb and trunk dynamics remains unexplored. Future research should explore the influence of landing direction and the biomechanical contributions of the upper limbs and trunk, as these factors may significantly affect joint loading and injury risk [33].

Conclusion

This study, based on Statistical Parametric Mapping, identified greater hip abduction, knee external rotation, and ankle internal rotation moments on the non-dominant side, along with increased vertical ground reaction forces at higher landing heights, as key contributors to elevated joint loading. These findings suggest that neuromuscular coordination, postural control, and movement symmetry may be critical in mitigating injury risks during high-impact landing tasks. Therefore, future training strategies could consider improving motor control and functional strength, particularly in the non-dominant limb, to enhance stability and optimize landing performance. Regulating landing height and optimizing landing mechanics may further reduce injury risk. Future research should integrate these insights with in-depth biomechanical analyses to develop evidence-based training protocols and injury prevention strategies, ultimately improving both performance and safety in rope skipping.

Declaration of competing interest

The authors declare no conflicts of interest, financial or personal, that could have influenced the research and findings.

Data availability

Data will be made available on request.

Acknowledgements

The authors thank all participants for their valuable contributions and the Biomechanics Laboratory at Yanshan University for providing the experimental facilities that enabled this research.

References

1. Lin Z, Zongyu Z, et al.. High-fiber diet and rope-skipping benefit cardiometabolic health and modulate gut microbiota in young adults: a randomized controlled trial. Food Res Int. 2023;173: 113421. https://doi.org/10.1016/j.foodres.2023.113421.

2. Xu D, Jiang X, Cen X, Baker JS, Gu Y. Single-leg landings following a volleyball spike may increase the risk of anterior cruciate ligament injury more than landing on both-legs. Appl Sci. 2020;11: 130. https://doi.org/10.3390/app11010130.

3. Zaheri RM, Majlesi M, Fatahi A. The effects of fatigue on the relationship between ankle angle at initial contact and the knee and hip joints in landing: Assessing the risk of ACL injury. Gait Posture. 2024;113: 462-467. https://doi.org/10.1016/j.gaitpost.2024.07.299.

4. Xu D, Zhou H, Wang M, Ma X, Gusztav F, Chon TE, et al.. Contribution of ankle motion pattern during landing to reduce the knee-related injury risk. Comput Biol Med. 2024;180: 108965. https://doi.org/10.1016/j.compbiomed.2024.108965.

5. Zhou SN. Contributions of lower extremity joints to energy dissipation during landings. Med Sci Sports Exerc. 2000;32: 812-819. https://doi.org/10.1097/00005768-200004000-00014.

6. Polk JD, Stumpf RM, Rosengren KS. Limb dominance, foot orientation and functional asymmetry during walking gait. Gait Posture. 2017;52: 140-146. https://doi.org/10.1016/j.gaitpost.2016.11.028.

7. Gabbard C, Hart S. A question of foot dominance. J Gen Psychol. 1996;123: 289-296. https://doi.org/10.1080/00221309.1996.9921281.

8. Hart NH, Newton RU, Weber J, Spiteri T, Rantalainen T, Dobbin M, et al.. Functional basis of asymmetrical lower-body skeletal morphology in professional Australian rules footballers. J Strength Cond Res. 2020;34: 791-799. https://doi.org/10.1519/JSC.0000000000002841.

9. Dos'Santos T, Thomas C, Jones PA. Assessing interlimb asymmetries: Are we heading in the right direction? Strength Cond J. 2021;43: 91-100. https://doi.org/10.1519/SSC.0000000000000590.

10. Krosshaug T, Nakamae A, Boden BP, Engebretsen L, Smith G, Slauterbeck JR, et al.. Mechanisms of anterior cruciate ligament injury in basketball: video analysis of 39 cases. Am J Sports Med. 2007;35: 359-367. https://doi.org/10.1177/0363546506293899.

11. Laughlin WA, Weinhandl JT, Kernozek TW, Cobb SC, Keenan KG, O'Connor KM. The effects of single-leg landing technique on ACL loading. J Biomech. 2011;44: 1845-1851. https://doi.org/10.1016/j.jbiomech.2011.04.010.

12. Leporace G, Pereira GR, Nadal J, Batista LA. Differences in time–frequency representation of lower limbs myoelectric activity during single and double leg landing in male athletes. J Electromyogr Kinesiol. 2011;21: 506-511. https://doi.org/10.1016/j.jelekin.2010.12.001.

13. Wattananon P, Kongoun S, Chohan A, Richards J. The use of statistical parametric mapping to determine altered movement patterns in people with chronic low back pain. J Biomech. 2023;153: 111601. https://doi.org/10.1016/j.jbiomech.2023.111601.

14. Pataky TC. Generalized n-dimensional biomechanical field analysis using statistical parametric mapping. J Biomech. 2010;43: 1976-1982. https://doi.org/10.1016/j.jbiomech.2010.03.008.

15. Pataky TC, Robinson MA, Vanrenterghem J. Vector field statistical analysis of kinematic and force trajectories. J Biomech. 2013;46: 2394-2401. https://doi.org/10.1016/j.jbiomech.2013.07.031.

16. Pataky TC, Vanrenterghem J, Robinson MA. Zero-vs. one-dimensional, parametric vs. non-parametric, and confidence interval vs. hypothesis testing procedures in one-dimensional biomechanical trajectory analysis. J Biomech. 2015;48: 1277-1285. https://doi.org/10.1016/j.jbiomech.2015.02.051.

17. Penny WD, Friston KJ, Ashburner JT, Kiebel SJ, Nichols TE. Statistical parametric mapping: the analysis of functional brain images. Elsevier. 2011;: . https://doi.org/10.1016/B978-0-12-372560-8.X5000-1.

18. Castro MP, Pataky TC, Sole G, Vilas-Boas JP. Pooling sexes when assessing ground reaction forces during walking: Statistical Parametric Mapping versus traditional approach. J Biomech. 2015;48: 2162-2165. https://doi.org/10.1016/j.jbiomech.2015.05.027.

19. Faul F, Erdfelder E, Lang AG, Buchner A. G* Power 3: A flexible statistical power analysis program for the social, behavioral, and biomedical sciences. Behav Res Methods. 2007;39: 175-191. https://doi.org/10.3758/BF03193146.

20. Yeow CH, Lee PVS, Goh JCH. Effect of landing height on frontal plane kinematics, kinetics and energy dissipation at lower extremity joints. J Biomech. 2009;42: 1967-1973. https://doi.org/10.1016/j.jbiomech.2009.05.017.

21. Monfort-Torres G, García-Massó X, Skýpala J, Blaschová D, Estevan I. Coordination and coordination variability during single-leg drop jump landing in children. Hum Mov Sci. 2024;96: 103251. https://doi.org/10.1016/j.humov.2024.103251.

22. de Oliveira I, Stoelben KJ, Tulius ES, de Pereira Ferreira V, Carpes FP. Strength and clinical test combinations enhance predictions of sagittal and frontal plane biomechanics in single-leg landing. Phys Ther Sport. 2024;69: 1-7. https://doi.org/10.1016/j.ptsp.2024.06.008.

23. Cappozzo A, Catani F, Della Croce U, Leardini A. Position and orientation in space of bones during movement: anatomical frame definition and determination. Clin Biomech. 1995;10: 171-178. https://doi.org/10.1016/0268-0033(95)91394-T.

24. Kristianslund E, Krosshaug T, Van den Bogert AJ. Effect of low pass filtering on joint moments from inverse dynamics: implications for injury prevention. J Biomech. 2012;45: 666-671. https://doi.org/10.1016/j.jbiomech.2011.12.011.

25. Lenz AL, Strobel MA, Anderson AM, Fial AV, MacWilliams BA, Krzak JJ, Kruger KM. Assignment of local coordinate systems and methods to calculate

tibiotalar and subtalar kinematics: a systematic review. J Biomech. 2021;120: 110344. https://doi.org/10.1016/j.jbiomech.2021.110344.

26. Elvin NG, Elvin AA, Arnoczky SP, Torry MR. The correlation of segment accelerations and impact forces with knee angle in jump landing. J Appl Biomech. 2007;23: 203-212. https://doi.org/10.1055/s-2007-965021.

27. Chappell JD, Yu B, Kirkendall DT, Garrett WE. A comparison of knee kinetics between male and female recreational athletes in stop-jump tasks. Am J Sports Med. 2002;30: 261-267. https://doi.org/10.1177/03635465020300021901.

28. Wakabayashi K, Ogasawara I, Suzuki Y, Nakata K, Nomura T. Exploring pre-impact landing kinematics associated with increase and decrease in the anterior cruciate ligament injury risk. J Biomech. 2022;145: 111382. https://doi.org/10.1016/j.jbiomech.2022.111382.

29. Ali N, Robertson DGE, Rouhi G. Sagittal plane body kinematics and kinetics during single-leg landing from increasing vertical heights and horizontal distances: Implications for risk of non-contact ACL injury. Knee. 2014;21: 38-46. https://doi.org/10.1016/j.knee.2012.12.003.

30. Devita P, Skelly WA. Effect of landing stiffness on joint kinetics and energetics in the lower extremity. Med Sci Sports Exerc. 1992;24: 108-115. https://doi.org/

31. Yu B, Lin CF, Garrett WE. Lower extremity biomechanics during the landing of a stop-jump task. Clin Biomech. 2006;21: 297-305. https://doi.org/10.1016/j.clinbiomech.2005.11.003.

32. Leppänen M, Pasanen K, Krosshaug T, Kannus P, Vasankari T, Kujala UM, et al.. Sagittal plane hip, knee, and ankle biomechanics and the risk of anterior cruciate ligament injury: a prospective study. Orthop J Sports Med. 2017;5: 2325967117745487. https://doi.org/10.1177/2325967117745487.

33. Shimokochi Y, Ambegaonkar JP, Meyer EG, Lee SY, Shultz SJ. Changing sagittal plane body position during single-leg landings influences the risk of non-contact anterior cruciate ligament injury. Knee Surg Sports Traumatol Arthrosc. 2013;21: 888-897. https://doi.org/10.1007/s00167-012-2011-9.

Supporting information

S1 Fig..tif **Fig. 1. Participants performing the single-leg landing task from two different heights (30 cm and 45 cm).** Note: Participants stood on the jumping box with one leg lifted, then leaned forward and landed on the test leg while keeping their hands on their hips. The two heights correspond to the experimental conditions used for data collection.

S2 Fig..tif **Fig.2. Single leg drop test manoeuvre.** Note：①The participant stands on the jumping box with the test leg suspended in the air and both hands placed on the hips, preparing to descend. ②The test leg begins to descend, and the body leans forward, utilizing inertia to approach the ground. ③The test leg makes contact with the ground, maintaining a stable posture, completing the test.

S3 Fig..tif **Fig. 3. Qualisys 3D Motion Capture System and Kistler 3D Force Stage.** Note: The test apparatus included: a Qualisys 3D motion capture system to capture motion in space (A), a Kistler 3D force table to measure changes in force (B), and an experimental test site (C).

S4 Fig..tif **Fig. 4. Landed buffer phase.** Note: The landing cushioning step is defined as the moment from the initial foot contact with the force platform to the maximum knee flexion angle, A: the moment from the initial foot contact with the force platform, B: the moment from the maximum knee flexion angle, C: the division of the landing cushioning phase.

S5 Fig..tif **Fig. 5. Comparison of vertical GRF SPM{F} in the landing cushion phase for different side and drop heights.** Note: Mean ensemble curves for vGRF (A) and their respective SPM{F} outputs (B, C, D) from 0 to 100% of stance phase. The dashed horizontal line on the SPM{F} outputs indicates the critical threshold boundary. The grey, shaded regions indicates the significant supra-threshold clusters representing statistically significant differences.

S6 Fig..tif **Fig. 6. Comparison of hip moments SPM{F} during the landing cushion phase for different side differences and drop heights.** Note: Mean ensemble curves for 3D hip joint moment (A) and their respective SPM{F} outputs (B, C, D) from 0 to 100% of stance phase. The dashed horizontal line on the SPM{F} outputs indicates the critical threshold boundary. The grey, shaded regions indicates the significant supra-threshold clusters representing statistically significant differences. Positive sagittal, frontal, and transverse plane knee joint kinetics represent internally applied knee extension/adduction/internal rotation moments.

S7 Fig..tif **Fig. 7. Comparison of knee moments SPM{F} during the landing cushion phase for different side and drop heights.** Note: Mean ensemble curves for 3D knee joint moment (A) and their respective SPM{F} outputs (B, C, D) from 0 to 100% of stance phase. The dashed horizontal line on the SPM{F} outputs indicates the critical threshold boundary. The grey, shaded regions indicates the significant supra-threshold clusters representing statistically significant differences. Positive sagittal, frontal, and transverse plane knee joint kinetics represent internally applied knee extension/adduction/internal rotation moments.

S8 Fig..tif **Fig. 8. Comparison of ankle moment SPM{F} during the landing cushioning phase for different side and drop heights.** Note: Mean ensemble curves for 3D ankle joint moment (A) and their respective SPM{F} outputs (B, C, D) from 0 to 100% of stance phase. The dashed horizontal line on the SPM{F} outputs indicates the critical threshold boundary. The grey, shaded regions indicates the significant supra-threshold clusters representing statistically significant differences. Positive sagittal, frontal, and transverse plane knee joint kinetics represent

internally applied knee extension/adduction/internal rotation moments.

S1 Table..pdf **Table 1. Comparison of vertical GRF peaks during the landing cushion phase for different side groups and drop heights（BW）.** Note: Mean (Standard difference), *:There was a significant difference between D30 and D45.

S2 Table..pdf **Table 2. Comparison of the peak moments of the lower limb joints during the landing cushioning phase by side and height of fall.** Note: Joint Moment (Peak Joint Moment), Mean (Standard difference), *: Significant difference between dominant and non-dominant side, #: There was a significant difference between D30 and D45.

S1 File..zip **Original data**

 Click here to access/download;Figure;Fig 7.docx

Fig. 7. Comparison of knee moments SPM{F} during the landing cushion phase for different side and drop heights.

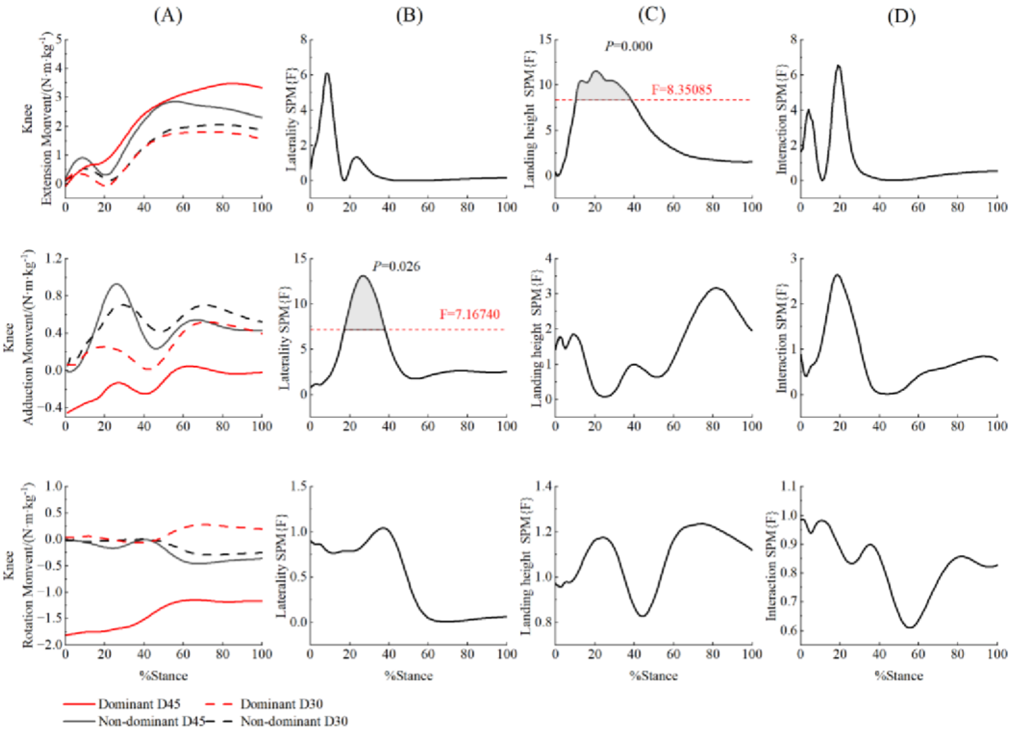

Note: Mean ensemble curves for 3D knee joint moment (A) and their respective SPM{F} outputs (B, C, D) from 0 to 100% of stance phase. The dashed horizontal line on the SPM{F} outputs indicates the critical threshold boundary. The grey, shaded regions indicates the significant supra-threshold clusters representing statistically significant differences. Positive sagittal, frontal, and transverse plane knee joint kinetics represent internally applied knee extension/adduction/internal rotation moments.

S8 Fig. Click here to access/download;Figure;Fig 8.docx

Fig. 8. Comparison of ankle moment SPM{F} during the landing cushioning phase for different side and drop heights.

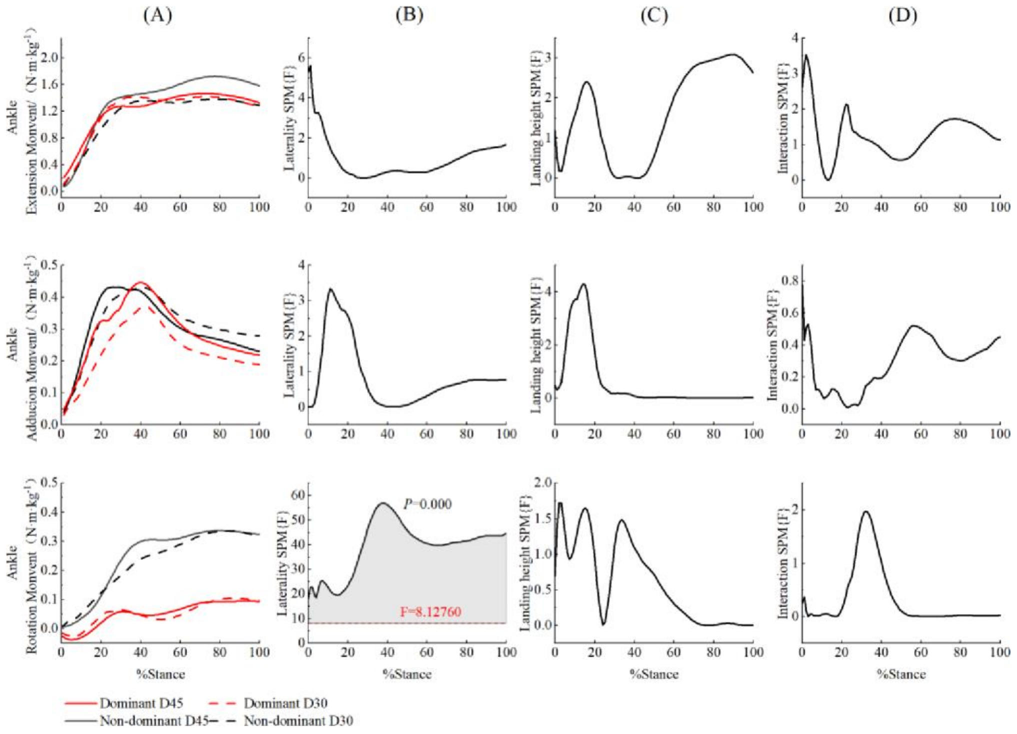

Note: Mean ensemble curves for 3D ankle joint moment (A) and their respective SPM{F} outputs (B, C, D) from 0 to 100% of stance phase. The dashed horizontal line on the SPM{F} outputs indicates the critical threshold boundary. The grey, shaded regions indicates the significant supra-threshold clusters representing statistically significant differences. Positive sagittal, frontal, and transverse plane knee joint kinetics represent internally applied knee extension/adduction/internal rotation moments.

Click here to access/download;Figure;Fig 5.docx

Fig. 5. Comparison of vertical GRF SPM{F} in the landing cushion phase for different side and drop heights.

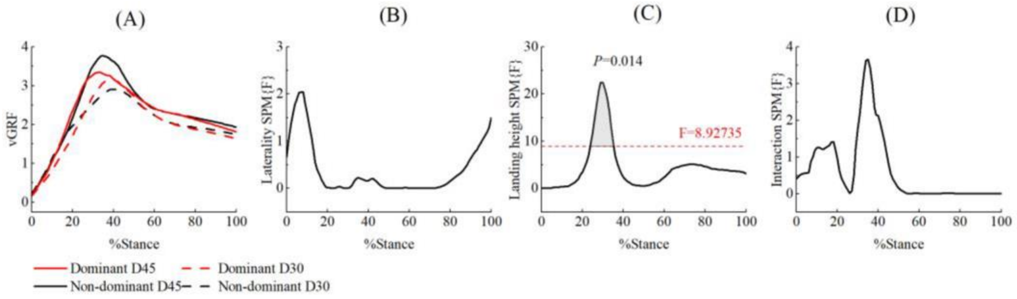

Note: Mean ensemble curves for vGRF (A) and their respective SPM{F} outputs (B, C, D) from 0 to 100% of stance phase. The dashed horizontal line on the SPM{F} outputs indicates the critical threshold boundary. The grey, shaded regions indicates the significant supra-threshold clusters representing statistically significant differences.

 Click here to access/download;Figure;Fig 6.docx

Fig. 6. Comparison of hip moments SPM{F} during the landing cushion phase for different side differences and drop heights.

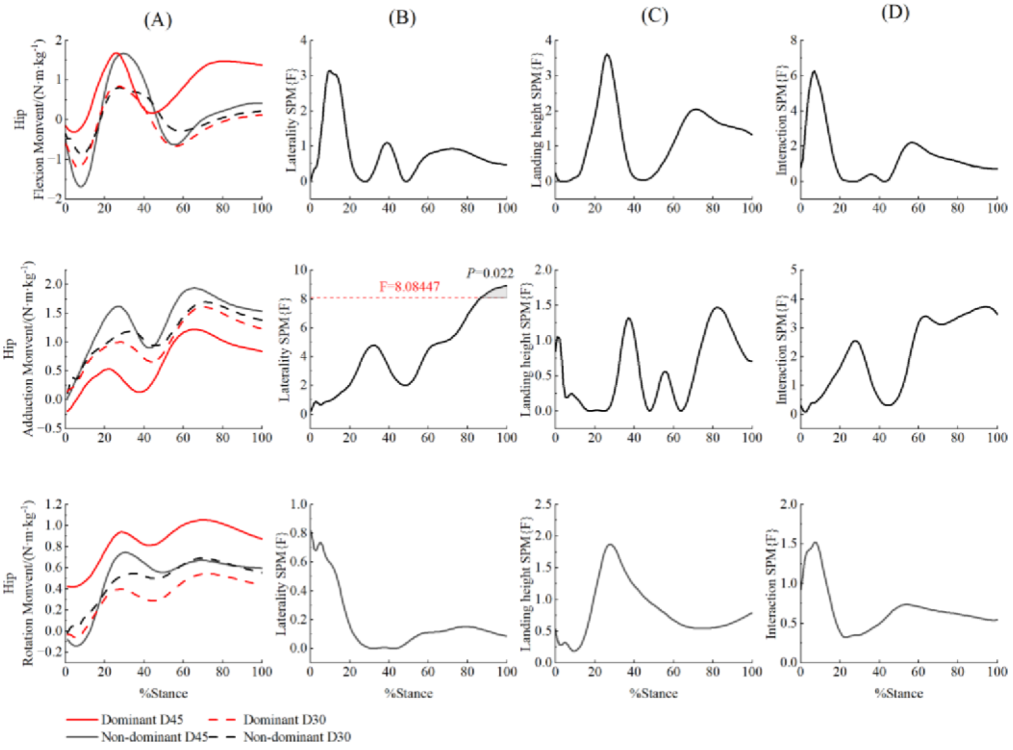

Note: Mean ensemble curves for 3D hip joint moment (A) and their respective SPM{F} outputs (B, C, D) from 0 to 100% of stance phase. The dashed horizontal line on the SPM{F} outputs indicates the critical threshold boundary. The grey, shaded regions indicates the significant supra-threshold clusters representing statistically significant differences. Positive sagittal, frontal, and transverse plane knee joint kinetics represent internally applied knee extension/adduction/internal rotation moments.

Click here to access/download;Figure;Fig 1.docx

Fig. 1. Participants performing the single-leg landing task from two different heights (30 cm and 45 cm).

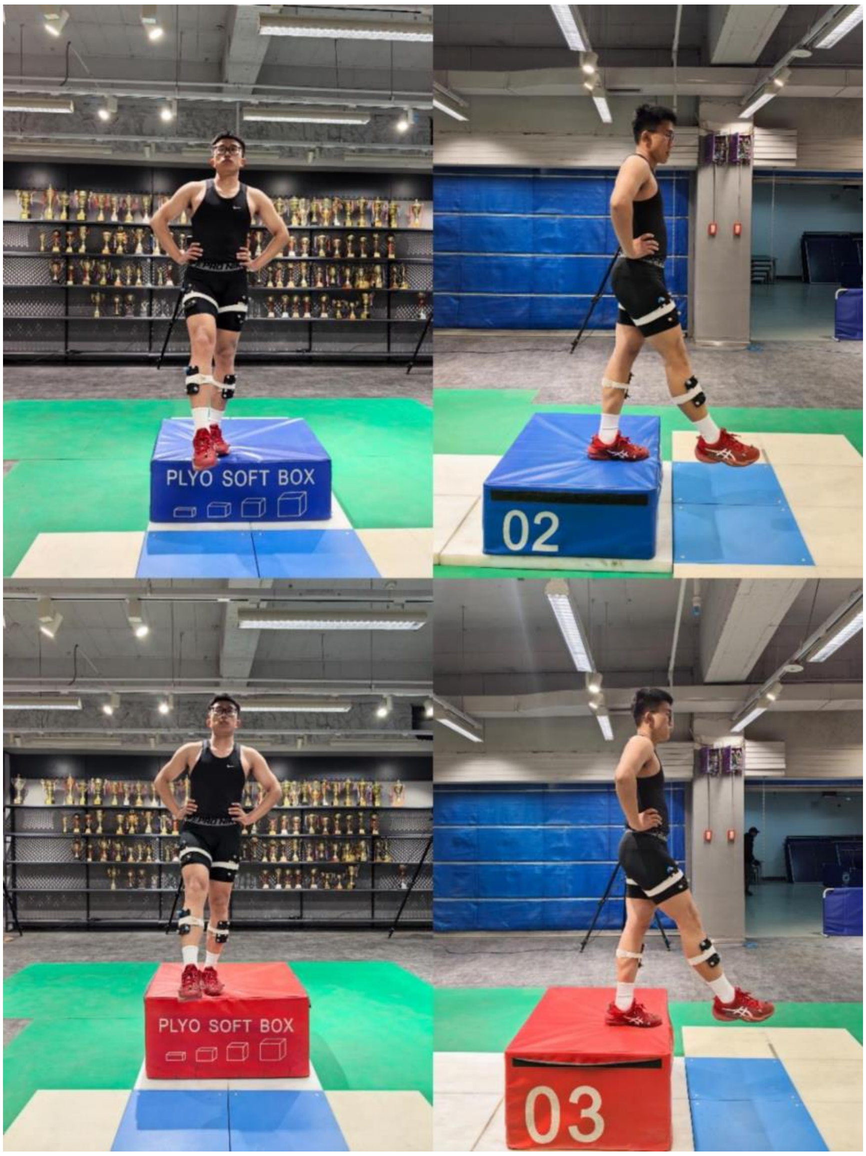

Note: Participants stood on the jumping box with one leg lifted, then leaned forward and landed on the test leg while keeping their hands on their hips. The two heights correspond to the experimental conditions used for data collection.

Fig.2. Single leg drop test manoeuvre

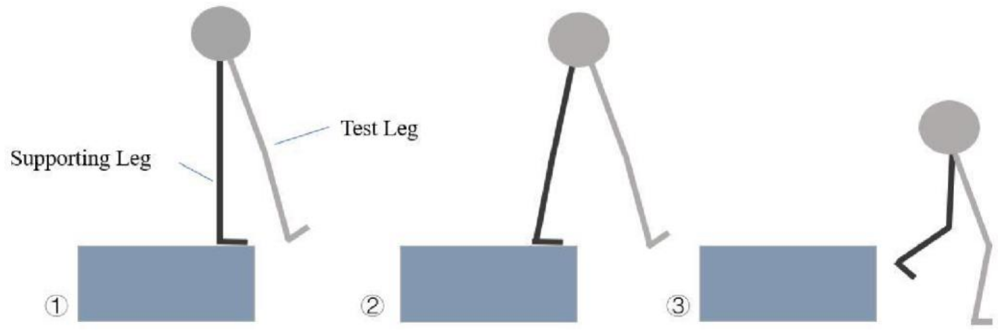

Note： □ The participant stands on the jumping box with the test leg suspended in the air and both hands placed on the hips, preparing to descend. □ The test leg begins to descend, and the body leans forward, utilizing inertia to approach the ground. □ The test leg makes contact with the ground, maintaining a stable posture, completing the test.

Click here to access/download;Figure;Fig 3.docx

Fig. 3. Qualisys 3D Motion Capture System and Kistler 3D Force Stage.

(A) (B) (C)

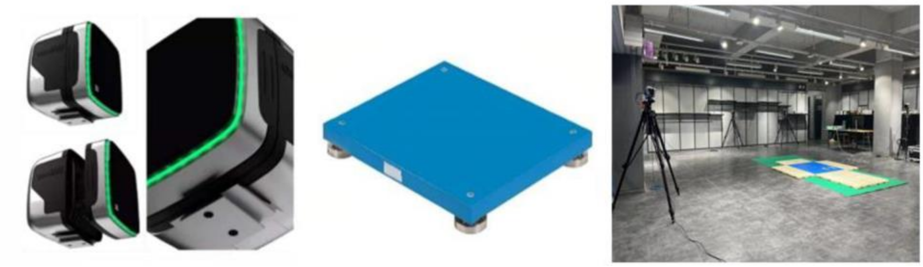

Note: The test apparatus included: a Qualisys 3D motion capture system to capture motion in space (A), a Kistler 3D force table to measure changes in force (B), and an experimental test site (C).

Click here to access/download;Figure;Fig 4.docx

Fig. 4. Landed buffer phase.

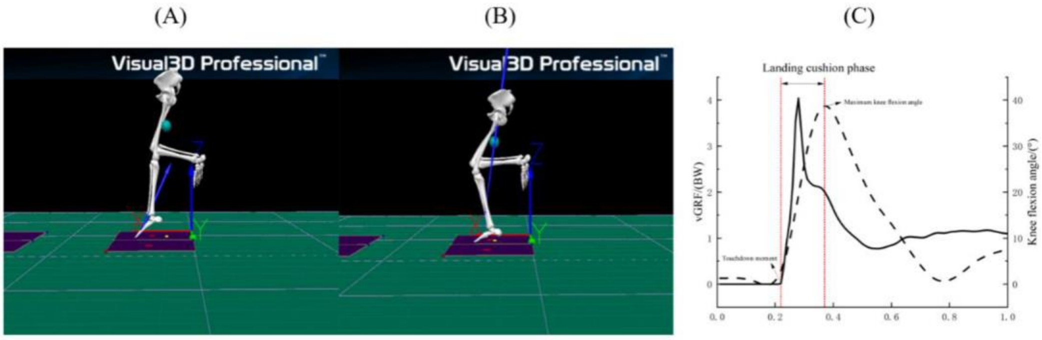

Note: The landing cushioning step is defined as the moment from the initial foot contact with the force platform to the maximum knee flexion angle, A: the moment from the initial foot contact with the force platform, B: the moment from the maximum knee flexion angle, C: the division of the landing cushioning phase.

S1 Table.

Click here to access/download
Supporting Information
Table 1.docx

S2 Table.

Click here to access/download
Supporting Information
Table 2.docx

Analysis of the Impact of Different Landing Heights and Lateralities on Lower Limb Joint Load Based on Statistical Parametric Mapping

Lian Duan[1¶], Yuan GAO[*¶], Feng GAO[*¶], Yang SUN[*&], Naiyuan Tian[2&]

[1] China Volleyball College, Beijing Sport University, Beijing, China.

[1] School of Physical Education, Yanshan University, Qinhuangdao, Hebei, China.

[*] School of Physical Education, Yanshan University, Qinhuangdao, Hebei, China.

[*] China Volleyball College, Beijing Sport University, Beijing, China.

[*] School of Physical Education, Yanshan University, Qinhuangdao, Hebei, China.

[2] School of Physical Education, Yanshan University, Qinhuangdao, Hebei, China.

* Corresponding author

Email: gaoyuan197812@126.com (YG)

Email: gaofeng202502@126.com (FG)

Email: syang0217@126.com (YS)

¶ These authors contributed equally to this work.

& These authors also contributed equally to this work.

Abstract

This study aimed to investigate the effects of landing height and limb laterality on lower limb joint loading using Statistical Parametric Mapping (SPM), thereby providing insights for injury prevention and optimizing performance in dynamic sports. Twenty male participants were assessed using the Qualisys 3D motion capture system and Kistler 3D force plates during landings from 30 cm and 45 cm on both dominant and non-dominant legs. No significant interaction effects between landing side and height were observed, but landing on the non-dominant leg increased the ankle joint internal rotation moment. The knee's abduction moment significantly increased during the 17%-37% landing phase, and the hip's abduction moment significantly increased during the 86%-100% phase. Higher landing height significantly increased vertical ground reaction force (GRF) during the mid-buffering phase (23%-35%). Landing on the dominant side was linked to an elevated risk of lower limb joint injuries due to increased abduction moments at the hip, external rotation moments at the knee, and internal rotation moments at the ankle. Furthermore, greater landing height correlated with higher vertical GRF, intensifying stress on the lower limbs and increasing injury risk. To reduce the risk of injury, training should focus on increasing strength and control of the non-dominant leg and controlling landing height.

Keywords: Statistical parameter mapping; Landing height; Lateralities; Lower limb joint loading

Introduction

In recent years, rope skipping has emerged as a widely adopted exercise modality, owing to its low cost, ease of learning, and cardiovascular benefits. Its effectiveness in improving both aerobic fitness and coordination has made it a favored exercise among fitness enthusiasts and recreational athletes alike [1]. Freestyle rope skipping, known for its diverse techniques and visually engaging performances, has gained widespread popularity. However, the complexity of these movements elevates the risk of injury, particularly during the landing phase. Recent studies highlight a notably high incidence of lower limb injuries (87.5%), with knee and ankle joints being most frequently affected, comprising approximately 80% of all injury cases [2-4]. Despite the human body's inherent structural symmetry, functional asymmetry, or laterality, is well-documented in lower limb biomechanics [5-6]. Excessive lateral laterality has been identified as a potential risk factor for non-contact joint injuries [7-8]. However, most rope skipping research assumes limb symmetry, primarily analyzing data from a single limb to assess landing-related injury risks [9-12]. Consequently, there is limited understanding of the biomechanical differences between dominant and non-dominant limbs during single-leg landing in rope skipping, highlighting a critical gap in the literature.

Statistical Parametric Mapping (SPM), based on random field theory, utilizes topological analysis to examine dynamic changes and interrelationships in time series data, enhancing the robustness of biomechanical conclusions, as indicated by

Wattananon et al. [13] and Pataky et al. [14-16]. While traditional zero-dimensional statistical methods can identify feature differences, they often fail to capture dynamic patterns in time series data, as highlighted by previous studies [16-18]. To overcome these limitations, this study aimed to systematically analyze the effects of landing height and limb lateralities on lower limb joint loading using Statistical Parametric Mapping (SPM). Specifically, the study focused on biomechanical differences between the dominant and non-dominant limbs during landings from different heights and their subsequent impact on joint loading. By addressing this gap in the literature, the study aims to provide scientific evidence for injury prevention in dynamic activities such as rope skipping and to optimize training strategies.

Materials and methods

Participants

Study participants were recruited between 15 April 2024 and 23 April 2024 through campus bulletin board postings and social media platforms targeting university students at Yanshan University, Qinhuangdao, Hebei Province. The sample size was determined using G*Power software with the following parameters: effect size f = 0.4, α error probability = 0.05, power (1-β) = 0.8, number of groups = 1, number of measurements = 2, and correlation among repeated measures = 0.5. The minimum

required sample size was calculated to be 15 participants [19]. To account for potential data loss due to movement failure, marker displacement, or invalid data, a 30% redundancy was added, Thus, 20 male rope-skipping enthusiasts participated in this study (age: 19.75 ± 0.86 years, height: 181.24 ± 4.62 cm, body mass：72.21± 7.66 kg, BMI: 22.06 ± 2.94 kg/m²). Data collection was completed during a 12-day experimental period following recruitment.

All participants were recruited based on the following inclusion criteria: (1) no congenital lower extremity deformities or acquired musculoskeletal disorders; (2) no history of lower extremity joint injuries within the past 6 months; and (3) sufficient physical ability to perform the task of stair walking. Exclusion criteria included (1) a history of lower extremity surgery; (2) use of medications affecting neuromuscular function within the past 12 months; (3) the presence of neuromuscular, psychologic, or cardiovascular disorders; and (4) the presence of acute pain symptoms during the test. All procedures were in accordance with the Declaration of Helsinki. Participants were fully informed of the purpose and procedures of the study and written informed consent was obtained. The study protocol was approved by the Ethics Committee of Qinhuangdao First Hospital.

The determination of the dominant side was conducted through a kicking test, where participants were asked to kick with both feet. The foot that achieved the greater kicking distance was identified as the dominant side [7], [20].

Single-leg landing test

Participants were instructed to place their hands on their hips and stand 5 cm from the force platform, with jumping boxes positioned at heights of 30 cm and 45 cm. Upon receiving a "start" command, participants lifted their test leg and held it in the air. They then leaned forward and descended by inertia, landing on the test foot to maintain posture and stability [2], [21]. Participants were required to focus visually on a target displayed on a blank wall in front of them throughout the test. Prior to the formal test, participants underwent a 6-minute familiarization process, including a movement demonstration and practice trials. Additionally, a 10-minute warm-up was conducted, consisting of 5 minutes of jogging and dynamic stretching, followed by 5 minutes of light jumping and landing exercises to prepare participants for the task. Trials were repeated if participants touched the ground with the non-test leg or used their arms for balance (Fig. 1 and 2) [22].

Data collection

During trials, participants wore study-specific tights, and after warming up, 20 infrared reflective markers were placed on anatomical landmarks according to the CAST (Calibrated Anatomical Systems Technique) lower limb model, which employs static calibration and marker tracking to establish joint coordinate systems while minimizing soft tissue artifacts [23]. All markers were applied by the same

experienced and professionally trained researcher, following standardized procedures and anatomical landmark definitions to ensure data accuracy and consistency. Following the collection of static model data, eight markers were removed from the medial and lateral sides of the knee and ankle joints. Participants performed at least three complete movements during trials, and kinematic and kinetic data were synchronously captured using the Qualisys 3D motion capture system (8 cameras, 200 Hz) and a Kistler 3D force platform (2000 Hz) during landing (Fig. 3).

Data processing

In this study, the landing cushion phase (the moment of contact of the test leg on the ground up to the maximum angle of knee flexion of the supporting leg) of the drop jump movement was recorded. The raw data were pre-processed using the QTM software supplied with the Qualisys system, and hip, thigh, calf, and foot coordinate systems were established based on the CAST lower limb model, using infrared reflective markers to create a simulation of the subject's lower limb. Kinematic data were subsequently imported into Visual 3D software for further processing. A fourth-order Butterworth low-pass filter with a cutoff frequency of 12 Hz was applied to smooth the data, as described by Kristianslund et al. [24], Lenz et al. [25] and Monfort-Torres et al. [21]. Joint moments for ankle plantarflexion, knee flexion, and hip flexion (N•m•kg) were defined as positive (+) values, while ankle dorsiflexion, knee extension, and hip extension moments were defined as negative (−) values.

Ground reaction forces (N) and joint moments (N•m•kg) Lenz et al. [25] were calculated through inverse dynamics (Fig. 4).

Statistical analysis

This study analyzes biomechanical indicators related to lower limb joint loads during the landing cushioning phase, defined as the period from initial ground contact by the supporting leg to maximum knee flexion. Joint moments were normalized to 101 data points across the landing phase for precise temporal resolution. Mean value curves were generated to compare dynamic variations in joint load related parameters. A two-way ANOVA was employed to examines the effects of side and landing height on peak vertical GRF and 3D joint moments during the cushioning phase, with normality and homogeneity of variance verified. SPM{F} analysis assessed side and landing height effects on vertical GRF and 3D joint moment time series. Statistical significance was set at $p < 0.05$. Analyses were performed with SPSS 23.0 and Origin 2022.

Results

Two-way ANOVA and SPM{F} analyses were performed to examine the effects of laterality (dominant vs non-dominant) and landing height (30 cm vs 45 cm) on lower extremity joint loads. The results showed that there was no significant interaction between laterality and height (p > 0.05). This suggests that these two factors

independently affect joint loads and have no combined effect.

Ground reaction forces in the landing cushioning phase

The two-way ANOVA results indicated that the peak vertical GRF during the landing cushioning phase was significantly greater for the 45 cm drop height (4.32 BW) compared to the 30 cm drop height (3.48 BW), independent of the side factor, with statistical significance denoted by $p < 0.001$ and F = 30.914 (Table 1).

Table 1. Comparison of vertical GRF peaks during the landing cushion phase for different side groups and drop heights（BW）

	**D30**	**D45**
**Non-dominant**	3.47±0.48$^{\#}$	4.31±0.64
**Dominant**	3.49±0.58	4.34±0.67

Note: Mean (Standard difference), *:There was a significant difference between D30 and D45.

The SPM{F} analysis revealed that during the 23% to 35% of the landing cushioning phase, the vertical GRF for the 45 cm drop height was significantly greater than that for the 30 cm drop height, regardless of the side factor, with $p = 0.004$ and F = 8.927 (Fig. 5).

Lower limb kinetic characteristics during the landing cushioning phase

The results of the two-way ANOVA indicate that during the landing cushioning phase,

the peak dorsiflexion moment of the ankle joint (0.99 N•m•kg^{-1} vs. 1.64 N•m•kg^{-1}, p = 0.01, F = 6.901), the peak internal rotation moment of the ankle joint (0.11 N•m•kg^{-1} vs. 0.37 N•m•kg^{-1}, p < 0.001, F = 47.348), the peak abduction moment of the knee joint (0.36 N•m•kg^{-1} vs. 0.88 N•m•kg^{-1}, p = 0.006, F = 7.834), and the peak external rotation moment of the knee joint (0.19 N•m•kg^{-1} vs. -0.41 N•m•kg^{-1}, p < 0.001, F = 58.042), were significantly greater on the non-dominant side compared to the dominant side. In addition, irrespective of the side factor, the peak flexion moment of the knee joint when landing from a 45 cm height was significantly greater (2.66 N•m•kg^{-1} vs. 2.03 N•m•kg^{-1}, p = 0.003, F = 9.132) than when landing from a 30 cm height (Table 2).

Table 2. Comparison of the peak moments of the lower limb joints during the landing cushioning phase by side and height of fall.

Peak moment (N·m·kg^{-1})	Dominant		Non-dominant		Interaction effect *p* value	Main effect of laterality *p* value	Main Effect of Height *p* value
	D30	D45	D30	D45			
Ankle dorsiflexion	0.95±1.39*	1.04±1.51	1.65±0.52	1.62±0.49	0.797	0.010	0.916
Ankle valgus	0.46±0.36	0.60±0.45	0.49±0.41	0.52±0.34	0.538	0.849	0.337
Ankle internal rotation	0.11±0.15*	0.11±0.18	0.36±0.17	0.38±0.17	0.862	0.000	0.878
Knee flexion	1.90±1.12#	2.42±1.15	2.16±0.65	2.89±0.72	0.598	0.077	0.003
Knee valgus	0.20±0.71*	0.23±0.98	0.90±0.56	0.85±0.96	0.549	0.006	0.391
Knee internal rotation	0.22±0.33*	0.17±0.47	-0.30±0.22	-0.51±0.35	0.327	0.000	0.107
Hip flexion	0.52±2.21	0.74±3.17	0.57±1.72	2.05±2.14	0.239	0.204	0.117

**Hip valgus**	1.79±1.47	1.80±1.44	1.93±0.49	2.13±0.47	0.707	0.345	0.672
**Hip internal rotation**	0.61±0.31	0.64±0.57	0.78±0.30	0.83±0.46	0.968	0.065	0.658

Note: Joint Moment (Peak Joint Moment), Mean (Standard difference), *: Significant difference between dominant and non-dominant side, #: There was a significant difference between D30 and D45.

The SPM{F} analysis indicated that during the 86% to 100% of the landing cushioning phase, the peak hip abduction moment was significantly greater on the non-dominant side compared to the dominant side (($F = 8.084$, $p = 0.022$), irrespective of the height factor (Fig. 6). Similarly, during the 17% to 37% of the landing cushioning phase, the peak abduction moment of the knee joint was significantly greater on the non-dominant side (($F = 7.167$, $p = 0.026$), independent of height factor (Fig. 7). Throughout the entire landing cushioning phase, the ankle internal rotation moment on the non-dominant side was significantly greater than on the dominant side ($F = 8.127$, $p < 0.001$) (Fig. 8). In addition, during the 10% to 38% of the landing cushioning phase, the knee joint flexion moment was significantly greater for a 45 cm landing ($F = 8.350$, $p < 0.001$) than for a 30 cm landing, regardless of the side factor (Fig. 7).

Discussion and analysis

Previous studies have primarily focused on single-limb mechanics, assuming bilateral symmetry, which limits understanding of limb asymmetries in dynamic activities. Given the high incidence of lower limb injuries, examining these differences is crucial.

This study aimed to investigate the effects of landing height and limb lateralities on joint loading, addressing this gap and providing insights for injury prevention and performance optimization.

Comparative analysis of side differences during the landing cushioning phase

Two-way ANOVA showed no significant difference in peak 3D hip moments regarding side or height, but SPM{F} revealed higher hip abduction moments on the non-dominant side, reflecting the need for stability during maximum knee flexion and impact absorption [20]. During landing, increased hip abduction moment in the frontal plane contributes to maintaining postural stability through coordinated muscular control and compensatory movement strategies [20, 22]. During the landing cushioning phase, the non-dominant leg exhibited a more pronounced increase in abduction moment to compensate for uneven strength and stabilize overall posture. Increased hip abduction moment may shift the center of pressure laterally, leading to compensatory changes in adjacent joints and potentially increasing the risk of knee valgus and ACL injury [10], [26-28]. The larger hip abduction moment in the non-dominant leg during the landing cushioning phase may impact the knee joint within the kinetic chain. Additionally, while SPM{F} analysis showed a significant increase in the non-dominant leg's hip abduction moment, no significant difference in external rotation moment was observed, likely due to the conservative significance

threshold used in zero-dimensional statistical analyses to prevent Type I errors [18]. Previous research has shown that zero-dimensional models have limitations in deriving probabilistic inferences from one-dimensional dynamic data [15-[17]17]. Although one-dimensional analysis may miss variations in knee joint external rotation, zero-dimensional models might still reveal significant differences. The SPM{F} results show that the non-dominant ankle exhibited a higher internal rotation moment during landing. This is likely due to the ankle's complex anatomy, which is inherently more prone to deformation upon impact. To compensate, the body increased the internal rotation moment for stability. Furthermore, the non-dominant side, with relatively weaker muscle strength and less precise neural control, may require greater plantarflexion and internal rotation to maintain landing stability [9].

Comparative analysis of different heights during the landing cushioning phase

Both two-way ANOVA and SPM{F} analyses confirmed that a 45 cm jump height generated significantly higher vertical GRF than a 30 cm jump. This aligns with physical principles: greater landing height increases vertical velocity with constant mass, requiring a higher impulse to counter momentum change. Due to the body's limited impact absorption capacity, excess force translates into higher GRF, amplifying landing impact. This finding supports Ali et al. [29], revealing a significant increase in knee flexion moment during landing from 45 cm compared to

30 cm. Previous studies have established that the knee joint, as the primary weight-bearing and energy-absorbing joint, plays a crucial role in impact force absorption during the cushioning phase [2], [30]. Accordingly, our study showed that higher landing heights resulted in increased impact forces, placing greater load on the knee for absorption and cushioning [31]. This is consistent with Leppänen et al. [32], which reported a significant positive correlation between knee flexion moments and the risk of ACL injury. Specifically, for every 10 N·m increase in knee flexion moment, the risk of ACL injury increased by 21% (HR = 1.21, 95% CI: 1.04–1.40, p = 0.01) [2], [20]. To reduce injury risk, rope jumping participants should control landing heights and gradually increase them during training. Complementing this with strength and coordination exercises to enhance lower limb muscle and joint stability during high-intensity exercise.

Limitations

This study, while providing valuable insights, has notable limitations. Conducted in a controlled lab setting, it does not fully replicate real-world rope skipping conditions. The focus on lower limb cushioning during forward landings overlooks other landing strategies, and the role of upper limb and trunk dynamics remains unexplored. Future research should explore the influence of landing direction and the biomechanical contributions of the upper limbs and trunk, as these factors may significantly affect joint loading and injury risk [33].

Conclusion

This study, based on Statistical Parametric Mapping, identified greater hip abduction, knee external rotation, and ankle internal rotation moments on the non-dominant side, along with increased vertical ground reaction forces at higher landing heights, as key contributors to elevated joint loading. These findings suggest that neuromuscular coordination, postural control, and movement symmetry may be critical in mitigating injury risks during high-impact landing tasks. Therefore, future training strategies could consider improving motor control and functional strength, particularly in the non-dominant limb, to enhance stability and optimize landing performance. Regulating landing height and optimizing landing mechanics may further reduce injury risk. Future research should integrate these insights with in-depth biomechanical analyses to develop evidence-based training protocols and injury prevention strategies, ultimately improving both performance and safety in rope skipping.

Declaration of competing interest

The authors declare no conflicts of interest, financial or personal, that could have influenced the research and findings.

Data availability

Data will be made available on request.

Acknowledgements

The authors thank all participants for their valuable contributions and the Biomechanics Laboratory at Yanshan University for providing the experimental facilities that enabled this research.

References

1. Lin Z, Zongyu Z, et al.. High-fiber diet and rope-skipping benefit cardiometabolic health and modulate gut microbiota in young adults: a randomized controlled trial. Food Res Int. 2023;173: 113421. https://doi.org/10.1016/j.foodres.2023.113421.

2. Xu D, Jiang X, Cen X, Baker JS, Gu Y. Single-leg landings following a volleyball spike may increase the risk of anterior cruciate ligament injury more than landing on both-legs. Appl Sci. 2020;11: 130. https://doi.org/10.3390/app11010130.

3. Zaheri RM, Majlesi M, Fatahi A. The effects of fatigue on the relationship between ankle angle at initial contact and the knee and hip joints in landing: Assessing the risk of ACL injury. Gait Posture. 2024;113: 462-467. https://doi.org/10.1016/j.gaitpost.2024.07.299.

4. Xu D, Zhou H, Wang M, Ma X, Gusztav F, Chon TE, et al.. Contribution of ankle motion pattern during landing to reduce the knee-related injury risk. Comput Biol Med. 2024;180: 108965. https://doi.org/10.1016/j.compbiomed.2024.108965.

5. Zhou SN. Contributions of lower extremity joints to energy dissipation during landings. Med Sci Sports Exerc. 2000;32: 812-819. https://doi.org/10.1097/00005768-200004000-00014.

6. Polk JD, Stumpf RM, Rosengren KS. Limb dominance, foot orientation and functional asymmetry during walking gait. Gait Posture. 2017;52: 140-146. https://doi.org/10.1016/j.gaitpost.2016.11.028.

7. Gabbard C, Hart S. A question of foot dominance. J Gen Psychol. 1996;123: 289-296. https://doi.org/10.1080/00221309.1996.9921281.

8. Hart NH, Newton RU, Weber J, Spiteri T, Rantalainen T, Dobbin M, et al.. Functional basis of asymmetrical lower-body skeletal morphology in professional Australian rules footballers. J Strength Cond Res. 2020;34: 791-799. https://doi.org/10.1519/JSC.0000000000002841.

9. Dos'Santos T, Thomas C, Jones PA. Assessing interlimb asymmetries: Are we heading in the right direction? Strength Cond J. 2021;43: 91-100. https://doi.org/10.1519/SSC.0000000000000590.

10. Krosshaug T, Nakamae A, Boden BP, Engebretsen L, Smith G, Slauterbeck JR, et al.. Mechanisms of anterior cruciate ligament injury in basketball: video analysis of 39 cases. Am J Sports Med. 2007;35: 359-367. https://doi.org/10.1177/0363546506293899.

11. Laughlin WA, Weinhandl JT, Kernozek TW, Cobb SC, Keenan KG, O'Connor KM. The effects of single-leg landing technique on ACL loading. J Biomech. 2011;44: 1845-1851. https://doi.org/10.1016/j.jbiomech.2011.04.010.

12. Leporace G, Pereira GR, Nadal J, Batista LA. Differences in time–frequency representation of lower limbs myoelectric activity during single and double leg landing in male athletes. J Electromyogr Kinesiol. 2011;21: 506-511. https://doi.org/10.1016/j.jelekin.2010.12.001.

13. Wattananon P, Kongoun S, Chohan A, Richards J. The use of statistical parametric mapping to determine altered movement patterns in people with chronic low back pain. J Biomech. 2023;153: 111601. https://doi.org/10.1016/j.jbiomech.2023.111601.

14. Pataky TC. Generalized n-dimensional biomechanical field analysis using statistical parametric mapping. J Biomech. 2010;43: 1976-1982. https://doi.org/10.1016/j.jbiomech.2010.03.008.

15. Pataky TC, Robinson MA, Vanrenterghem J. Vector field statistical analysis of kinematic and force trajectories. J Biomech. 2013;46: 2394-2401. https://doi.org/10.1016/j.jbiomech.2013.07.031.

16. Pataky TC, Vanrenterghem J, Robinson MA. Zero-vs. one-dimensional, parametric vs. non-parametric, and confidence interval vs. hypothesis testing procedures in one-dimensional biomechanical trajectory analysis. J Biomech. 2015;48: 1277-1285. https://doi.org/10.1016/j.jbiomech.2015.02.051.

17. Penny WD, Friston KJ, Ashburner JT, Kiebel SJ, Nichols TE. Statistical parametric mapping: the analysis of functional brain images. Elsevier. 2011;: . https://doi.org/10.1016/B978-0-12-372560-8.X5000-1.

18. Castro MP, Pataky TC, Sole G, Vilas-Boas JP. Pooling sexes when assessing ground reaction forces during walking: Statistical Parametric Mapping versus traditional approach. J Biomech. 2015;48: 2162-2165. https://doi.org/10.1016/j.jbiomech.2015.05.027.

19. Faul F, Erdfelder E, Lang AG, Buchner A. G* Power 3: A flexible statistical power analysis program for the social, behavioral, and biomedical sciences. Behav Res Methods. 2007;39: 175-191. https://doi.org/10.3758/BF03193146.

20. Yeow CH, Lee PVS, Goh JCH. Effect of landing height on frontal plane kinematics, kinetics and energy dissipation at lower extremity joints. J Biomech. 2009;42: 1967-1973. https://doi.org/10.1016/j.jbiomech.2009.05.017.

21. Monfort-Torres G, García-Massó X, Skýpala J, Blaschová D, Estevan I. Coordination and coordination variability during single-leg drop jump landing in children. Hum Mov Sci. 2024;96: 103251. https://doi.org/10.1016/j.humov.2024.103251.

22. de Oliveira I, Stoelben KJ, Tulius ES, de Pereira Ferreira V, Carpes FP. Strength and clinical test combinations enhance predictions of sagittal and frontal plane biomechanics in single-leg landing. Phys Ther Sport. 2024;69: 1-7. https://doi.org/10.1016/j.ptsp.2024.06.008.

23. Cappozzo A, Catani F, Della Croce U, Leardini A. Position and orientation in space of bones during movement: anatomical frame definition and determination. Clin Biomech. 1995;10: 171-178. https://doi.org/10.1016/0268-0033(95)91394-T.

24. Kristianslund E, Krosshaug T, Van den Bogert AJ. Effect of low pass filtering on joint moments from inverse dynamics: implications for injury prevention. J Biomech. 2012;45: 666-671. https://doi.org/10.1016/j.jbiomech.2011.12.011.

25. Lenz AL, Strobel MA, Anderson AM, Fial AV, MacWilliams BA, Krzak JJ, Kruger KM. Assignment of local coordinate systems and methods to calculate

tibiotalar and subtalar kinematics: a systematic review. J Biomech. 2021;120: 110344. https://doi.org/10.1016/j.jbiomech.2021.110344.

26. Elvin NG, Elvin AA, Arnoczky SP, Torry MR. The correlation of segment accelerations and impact forces with knee angle in jump landing. J Appl Biomech. 2007;23: 203-212. https://doi.org/10.1055/s-2007-965021.

27. Chappell JD, Yu B, Kirkendall DT, Garrett WE. A comparison of knee kinetics between male and female recreational athletes in stop-jump tasks. Am J Sports Med. 2002;30: 261-267. https://doi.org/10.1177/03635465020300021901.

28. Wakabayashi K, Ogasawara I, Suzuki Y, Nakata K, Nomura T. Exploring pre-impact landing kinematics associated with increase and decrease in the anterior cruciate ligament injury risk. J Biomech. 2022;145: 111382. https://doi.org/10.1016/j.jbiomech.2022.111382.

29. Ali N, Robertson DGE, Rouhi G. Sagittal plane body kinematics and kinetics during single-leg landing from increasing vertical heights and horizontal distances: Implications for risk of non-contact ACL injury. Knee. 2014;21: 38-46. https://doi.org/10.1016/j.knee.2012.12.003.

30. Devita P, Skelly WA. Effect of landing stiffness on joint kinetics and energetics in the lower extremity. Med Sci Sports Exerc. 1992;24: 108-115. https://doi.org/

31. Yu B, Lin CF, Garrett WE. Lower extremity biomechanics during the landing of a stop-jump task. Clin Biomech. 2006;21: 297-305. https://doi.org/10.1016/j.clinbiomech.2005.11.003.

32. Leppänen M, Pasanen K, Krosshaug T, Kannus P, Vasankari T, Kujala UM, et al.. Sagittal plane hip, knee, and ankle biomechanics and the risk of anterior cruciate ligament injury: a prospective study. Orthop J Sports Med. 2017;5: 2325967117745487. https://doi.org/10.1177/2325967117745487.

33. Shimokochi Y, Ambegaonkar JP, Meyer EG, Lee SY, Shultz SJ. Changing sagittal plane body position during single-leg landings influences the risk of non-contact anterior cruciate ligament injury. Knee Surg Sports Traumatol Arthrosc. 2013;21: 888-897. https://doi.org/10.1007/s00167-012-2011-9.

Supporting information

S1 Fig..tif **Fig. 1. Participants performing the single-leg landing task from two different heights (30 cm and 45 cm).** Note: Participants stood on the jumping box with one leg lifted, then leaned forward and landed on the test leg while keeping their hands on their hips. The two heights correspond to the experimental conditions used for data collection.

S2 Fig..tif **Fig.2. Single leg drop test manoeuvre** Note：①The participant stands on the jumping box with the test leg suspended in the air and both hands placed on the hips, preparing to descend. ②The test leg begins to descend, and the body leans forward, utilizing inertia to approach the ground. ③The test leg makes contact with the ground, maintaining a stable posture, completing the test.

S3 Fig..tif **Fig. 3. Qualisys 3D Motion Capture System and Kistler 3D Force Stage** Note: The test apparatus included: a Qualisys 3D motion capture system to capture motion in space (A), a Kistler 3D force table to measure changes in force (B), and an experimental test site (C).

S4 Fig..tif **Fig. 4. Landed buffer phase** Note: The landing cushioning step is defined as the moment from the initial foot contact with the force platform to the maximum knee flexion angle, A: the moment from the initial foot contact with the force platform, B: the moment from the maximum knee flexion angle, C: the division of the landing cushioning phase.

S5 Fig..tif **Fig. 5. Comparison of vertical GRF SPM{F} in the landing cushion phase for different side and drop heights.** Note: Mean ensemble curves for vGRF (A) and their respective SPM{F} outputs (B, C, D) from 0 to 100% of stance phase. The dashed horizontal line on the SPM{F} outputs indicates the critical threshold boundary. The grey, shaded regions indicates the significant supra-threshold clusters representing statistically significant differences.

S6 Fig..tif **Fig. 6. Comparison of hip moments SPM{F} during the landing cushion phase for different side differences and drop heights.** Note: Mean ensemble curves for 3D hip joint moment (A) and their respective SPM{F} outputs (B, C, D) from 0 to 100% of stance phase. The dashed horizontal line on the SPM{F} outputs indicates the critical threshold boundary. The grey, shaded regions indicates the significant supra-threshold clusters representing statistically significant differences. Positive sagittal, frontal, and transverse plane knee joint kinetics represent internally applied knee extension/adduction/internal rotation moments.

S7 Fig..tif **Fig. 7. Comparison of knee moments SPM{F} during the landing cushion phase for different side and drop heights.** Note: Mean ensemble curves for 3D knee joint moment (A) and their respective SPM{F} outputs (B, C, D) from 0 to 100% of stance phase. The dashed horizontal line on the SPM{F} outputs indicates the critical threshold boundary. The grey, shaded regions indicates the significant supra-threshold clusters representing statistically significant differences. Positive sagittal, frontal, and transverse plane knee joint kinetics represent internally applied knee extension/adduction/internal rotation moments.

S8 Fig..tif **Fig. 8. Comparison of ankle moment SPM{F} during the landing cushioning phase for different side and drop heights.** Note: Mean ensemble curves for 3D ankle joint moment (A) and their respective SPM{F} outputs (B, C, D) from 0 to 100% of stance phase. The dashed horizontal line on the SPM{F} outputs indicates the critical threshold boundary. The grey, shaded regions indicates the significant supra-threshold clusters representing statistically significant differences. Positive sagittal, frontal, and transverse plane knee joint kinetics represent

internally applied knee extension/adduction/internal rotation moments.

S1 Table..pdf **Table 1. Comparison of vertical GRF peaks during the landing cushion phase for different side groups and drop heights（BW）** Note: Mean (Standard difference), *:There was a significant difference between D30 and D45.

S2 Table..pdf **Table 2. Comparison of the peak moments of the lower limb joints during the landing cushioning phase by side and height of fall.** Note: Joint Moment (Peak Joint Moment), Mean (Standard difference), *: Significant difference between dominant and non-dominant side, #: There was a significant difference between D30 and D45.

S1 File..zip **Original data**

Response to Reviewer #1

Thank you for your valuable suggestions, here are the specific changes we have made：

1. Errors in the text involving tenses and grammar have been corrected in accordance with your suggestions.

2. A new paragraph has been added to line 61 based on your suggestion.

3. Thank you for your valuable question regarding participant recruitment and quantity selection. In response, we have modified the original paragraph to read;
Study participants were recruited between 15 April 2024 and 23 April 2024 through campus bulletin board postings and social media platforms targeting university students at Yanshan University, Qinhuangdao, Hebei Province. The sample size was determined using G*Power software with the following parameters: effect size f = 0.4, α error probability = 0.05, power (1-β) = 0.8, number of groups = 1, number of measurements = 2, and correlation among repeated measures = 0.5. The minimum required sample size was calculated to be 15 participants [19]. To account for potential data loss due to movement failure, marker displacement, or invalid data, a 30% redundancy was added, Thus, 20 male rope-skipping enthusiasts participated in this study (age: 19.75 ± 0.86 years, height: 181.24 ± 4.62 cm, body mass：72.21± 7.66 kg, BMI: 22.06 ± 2.94 kg/m²). Data collection was completed during a 12-day experimental period following recruitment.

4. We have corrected the terminology to refer to "body mass" instead of "weight" to accurately reflect the use of kilograms (kg) as the unit of measurement.

5. In response to your question about the exclusion criteria for subjects, we have

added a detailed description of the inclusion and exclusion criteria in Section Participants. which reads:

All participants were recruited based on the following inclusion criteria: (1) no congenital lower extremity deformities or acquired musculoskeletal disorders; (2) no history of lower extremity joint injuries within the past 6 months; and (3) sufficient physical ability to perform the task of stair walking. Exclusion criteria included (1) a history of lower extremity surgery; (2) use of medications affecting neuromuscular function within the past 12 months; (3) the presence of neuromuscular, psychologic, or cardiovascular disorders; and (4) the presence of acute pain symptoms during the test.

6. In response to your question about subjects familiarizing themselves with the movements and warming up. We have added content notes to the "Single Leg Landing Test" section as follows:

Prior to the formal test, participants underwent a 6-minute familiarization process, including a movement demonstration and practice trials. Additionally, a 10-minute warm-up was conducted, consisting of 5 minutes of jogging and dynamic stretching, followed by 5 minutes of light jumping and landing exercises to prepare participants for the task.

7. Regarding your question: who placed the markers? what was their level of experience? We have added a description of the relevant content in the Data collection section, as follows:

All markers were applied by the same experienced and professionally trained researcher, following standardized procedures and anatomical landmark definitions to ensure data accuracy and consistency.

8. In response to your suggestion, added references to CAST lower extremity modeling.

[23] Cappozzo, A., Catani, F., Della Croce, U., & Leardini, A. (1995). Position and

orientation in space of bones during movement: anatomical frame definition and determination. Clinical biomechanics, 10(4), 171-178. https://doi.org/10.1016/0268-0033(95)91394-T.

9. Regarding your insightful question about data filtering.

In this study, a fourth-order Butterworth low-pass filter with a cutoff frequency of 12 Hz was mainly applied to smooth the trajectory data. The joint torque data were not filtered because they were derived using inverse dynamics, where the input data (including motion trajectory and filtered ground reaction force (GRF) data) were processed before calculation. Although the raw GRF data were collected using a high-precision Kistler force plate, the same 12 Hz low-pass filter was applied to the GRF data to ensure consistency with the frequency characteristics of the motion data, so the torque data itself did not need secondary filtering.

10. Regarding your question about the statement of Joint moments.

The moments mentioned in this study refer to joint moments, which are calculated by inverse dynamics methods, not internal net moments. Joint moments are calculated based on kinematic trajectories and ground reaction force (GRF) data.

11. Regarding the incorrect structure of the first sentence of the result section, I have modified it. The specific content is as follows:

Two-way ANOVA and SPM{F} analyses were performed to examine the effects of laterality (dominant vs non-dominant) and landing height (30 cm vs 45 cm) on lower extremity joint loads. The results showed that there was no significant interaction between laterality and height ($P > 0.05$). This suggests that these two factors independently affect joint loads and have no combined effect.

12. Added the *p*-values in Table 2 based on your suggestion, and added explanation for the peak moment in the table.

13. According to your suggestion, the problem to be solved and the purpose of the

study are explained before the discussion begins. The specific contents is as follows:

Previous studies have primarily focused on single-limb mechanics, assuming bilateral symmetry, which limits understanding of limb asymmetries in dynamic activities. Given the high incidence of lower limb injuries, examining these differences is crucial. This study aimed to investigate the effects of landing height and limb lateralities on joint loading, addressing this gap and providing insights for injury prevention and performance optimization.

14. Regarding the description of the positive correlation in the cited Leppänen article, it has been revised in the text as follows:

This is consistent with Leppänen et al. [32], which reported a significant positive correlation between knee flexion moments and the risk of ACL injury. Specifically, for every 10 N·m increase in knee flexion moment, the risk of ACL injury increased by 21% (HR = 1.21, 95% CI: 1.04–1.40, $p = 0.01$) [**Error! Bookmark not defined.**, 20].

Because Leppänen et al. reported hazard ratios (HR) rather than correlation coefficients (r). Due to methodological differences, the assessment of ACL injury risk is typically conducted using survival analysis and regression models; therefore, r values were not provided in their study.

Response to Reviewer #2

1. Reviewer #2: Suggestion: In the abstract, you use “knee's external abduction moment” (row 30), and “hip's external abduction moment” (row 31). There is no concept like “external abduction”. If you say that the moment is external, so how should we understand the “ankle joint internal rotation moment” (row 29)? Like internal moment or internal rotation in the ankle? Furthermore, you never mention them again in the text.

Author: Thank you for pointing out the problem of inconsistent and ambiguous terminology describing joint moments in the abstract. We agree that phrases such as “external abduction moment” are imprecise and may cause confusion. To clarify, we have revised the wording as follows: “The knee's abduction moment significantly increased during the 17%–37% landing phase, and the hip's abduction moment significantly increased during the 86%–100% phase.”

2. Reviewer #2: The purpose of the study is not clearly stated.

Author: Thank you for your helpful suggestions on clarifying the purpose of the study. In response, we have revised the opening sentence of the Abstract to explicitly state the aim of the study. In addition, we have updated the final paragraph of the Introduction to clearly articulate the specific objectives, research focus, and intended contributions of the work.

3. Reviewer #2: Your introduction section focuses entirely on the rope jumping process, while the study only includes landing from different heights, without a

preceding flight phase. Indeed this investigation could relate to many other “landing” activities (for example all kinds of jumps). A revision in this section is needed. You perform the experimental investigation with rope-skipping enthusiasts, not professionals. How often do these enthusiasts practice rope-skipping, and do they practice other sports, leading to different leg strengths?

Author: Thank you for your insightful comments regarding the focus of the introduction and the sample population studied. Regarding the focus on rope skipping, we acknowledge that the introduction may have emphasized this specific activity. However, the primary goal of the study was to explore landing mechanics, which are not limited to rope skipping but extend to various dynamic landing activities such as jumps from different heights. The focus on rope skipping enthusiasts was driven by the practical need to assess biomechanics in a commonly practiced activity where landing injuries are prevalent, thus providing relevant data for injury prevention and performance optimization in this context.

In terms of the sample population, we understand the importance of leg strength and the potential impact of training frequency on biomechanics. However, the participants in this study were specifically selected to represent rope skipping enthusiasts with regular training, and we did not explore additional factors such as cross-training or other sports. While the potential variability in leg strength could be considered in future research, the current study's aim was to focus on the biomechanical effects of landing height and limb lateralities, which are independent of the participants’ leg strength or training history. Future studies could expand this research by incorporating athletes with different training backgrounds or exploring variations in landing techniques across a broader range of activities.

Reviewer #2: During standard rope-skipping man jump approximately 15-30 sm in height. How did you determine your 30 and 45 sm?

Author: Thank you for your valuable comments regarding the selection of 30 cm and

45 cm as landing heights. In standard rope skipping, the vertical displacement during flight is relatively small (typically 15–30 cm), as noted. However, the heights of 30 cm and 45 cm were intentionally selected to simulate increased landing intensity conditions, which are commonly encountered in freestyle rope skipping or other jumping-related activities where athletes perform complex aerial movements before landing.

These controlled drop heights are widely used in biomechanical research to safely reproduce variable landing loads and assess their effects on joint kinetics and kinematics under standardized conditions. The use of 30 cm and 45 cm also aligns with prior studies analyzing single-leg or drop landings to provoke meaningful biomechanical responses, particularly in joint loading and ground reaction forces. Therefore, while these heights may exceed typical skipping jump heights, they provide a practical and validated model for investigating the effects of increased landing demand, which is relevant not only to rope skipping but to various athletic movements involving unilateral landings.

4. Reviewer #2: Please add a citation for “CAST lower limb model” and explain the abbreviation.

Author: Thank you for pointing out this important problemand we have made the following changes.

During trials, participants wore study-specific tights, and after warming up, 20 infrared reflective markers were placed on anatomical landmarks according to the CAST (Calibrated Anatomical Systems Technique) lower limb model, which employs static calibration and marker tracking to establish joint coordinate systems while minimizing soft tissue artifacts [23].

[23] Cappozzo, A., Catani, F., Della Croce, U., & Leardini, A. (1995). Position and orientation in space of bones during movement: anatomical frame definition and determination. Clinical biomechanics, 10(4), 171-178. https://doi.org/10.1016/0268-0033(95)91394-T.

5. Reviewer #2: In addition to photos of the equipment, you can also add one or some participants during the experiment.

Author: Thank you for your suggestion, to which we added images of the participants.

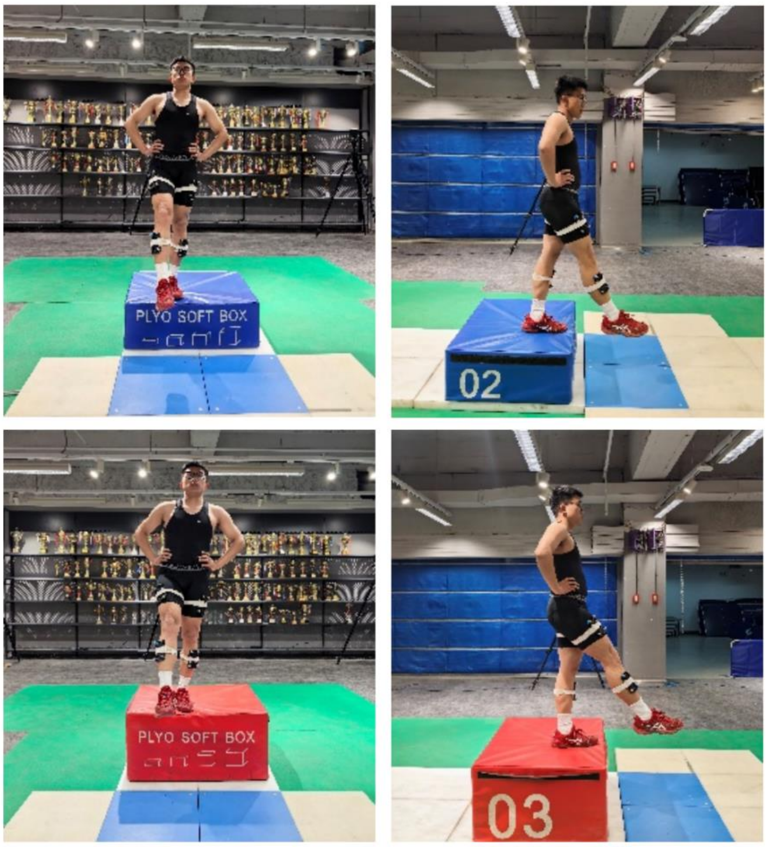

Fig. 3. Participants performing the single-leg landing task from two different heights (30 cm and 45 cm).

Note: Participants stood on the jumping box with one leg lifted, then leaned forward and landed on the test leg while keeping their hands on their hips. The two heights correspond to the experimental conditions used for data collection.

6. Reviewer #2: "This study analyzes biomechanical indicators …" (row 132). Which are they?

Author: We appreciate your attention to detail. The biomechanical indicators

analyzed in this study include vertical ground reaction force (vGRF) and three-dimensional joint moments of the hip, knee, and ankle during the landing cushioning phase. Specifically, the joint moments include:

Hip: flexion/extension, abduction/adduction, internal/external rotation

Knee: flexion/extension, varus/valgus, internal/external rotation

Ankle: plantarflexion/dorsiflexion, inversion/eversion, internal/external rotation

These indicators are described in detail in Section Statistical Analysis and summarized throughout the Results section (Tables 1–2 and Figures 4–7).

7. Reviewer #2: “The hip joint stabilizes the coronal plane by increasing abduction moments…” (row 229). This sentence structure is not correct. Hip joints do not stabilize any plane. Abduction movement is performed in that plane.

Author: Thank you for your insightful comments! We agree that the hip joint itself does not stabilize any anatomical plane. Rather, we intended to express that hip abduction torque, which occurs in the frontal plane, contributes to dynamic postural control during landing. We have revised the sentence accordingly to avoid misinterpretation.

During landing, increased hip abduction moment in the frontal plane contributes to maintaining postural stability through coordinated muscular control and compensatory movement strategies.

8. Reviewer #2: “Excessive hip abduction can shift the center of pressure outward, leading to compensatory changes in adjacent joints, increasing knee valgus and ACL injury risk ” (row 232). During drop landing, you do not observe “excessive hip abduction” which is over 50 degrees in the joint, but only increased abduction moment.

Author: Thank you for pointing this out. We agree that “excessive hip abduction” may be misleading in the context of our findings, as our study did not assess hip

abduction angles but rather observed increased hip abduction moments on the non-dominant side during landing. We have revised the sentence to accurately reflect this and avoid confusion between joint angle and joint moment.

The revised paragraph reads: Increased hip abduction moment may shift the center of pressure laterally, leading to compensatory changes in adjacent joints and potentially increasing the risk of knee valgus and ACL injury.

9. Reviewer #2: Rows 251-255. I recommend you 1) move it to the conclusion section; 2) do not be so confident in speculating the results with the need for "strength training ". Joint stability depends on bone shape congruency, capsule and ligament elasticity, muscle strength, neuromuscular control, fatigue. Better find some previous literature sources, and think in the field of coordination, and motor control.

Author: Thank you for the valuable feedback. We agree that joint stability is a multifactorial phenomenon influenced by not only muscle strength but also ligament properties, joint congruency, neuromuscular control, and fatigue. In response, the statement has been moved to the conclusion section as recommended.

10. Reviewer #2: Some citations are missing from the text like 20, 22, 23…

Thank you for pointing out the problem regarding missing citations. Upon careful review, we would like to clarify that reference 20 is cited in Section Participants of the manuscript. Reference 22 is included in Section Single-leg landing test. References 23 and 24 are cited in the Data collection and Data processing sections, respectively. Some of these references also appear later as cross-references to support relevant discussions. We have carefully checked the entire manuscript to ensure that all citations are accurate and consistently presented.

Thanks again for your valuable suggestions.
