# Supplementary material for: Analysis of the impact of different landing heights and lateralities on lower limb joint load based on statistical parametric mapping
Source: PLoS One. 2025 May 30;20(5):e0324656. doi: 10.1371/journal.pone.0324656 (PMC12124505; doi:10.1371/journal.pone.0324656)
Supplement: Attachment — Submitted filename: Response_to_Reviewers_auresp_2.docx [file pone.0324656.s005.docx]

**Response to Reviewer**

Thank you for your valuable suggestions, here are the specific changes we have made：

1. Errors in the text involving tenses and grammar have been corrected in accordance with your suggestions.
2. A fourth-order Butterworth low-pass filter with a cutoff frequency of 12 Hz was applied to smooth the data, as described by Kristianslund et al. [24], Lenz et al. [25] and Monfort-Torres et al. [21].
    Thank you for your valuable questions regarding the data smoothing process in this study. In response, we have modified the original paragraph as follows：

A fourth-order Butterworth low-pass filter with a cutoff frequency of 12 Hz was applied to smooth the trajectory data, as described by Kristianslund et al. [^[1]^], Lenz et al. [^[2]^] and Monfort-Torres et al. [21].

3. After reviewing the relevant literature, we have changed “joint moments” to “Net internal joint moments” according to your suggestion, thank you for pointing out the key issues.
4. During landing, increased[20] hip abduction moment in the frontal plane contributes to maintaining postural stability through coordinated muscular control and compensatory movement strategies[20, 22].

The original text has been modified in accordance with your suggestions: During landing, increased hip abduction moment contributes to maintaining postural stability through coordinated muscular control and compensatory movement strategies [20, 22].

5. This is likely due to the ankle's complex anatomy, which is inherently more prone to deformation upon impact.

Thank you for your suggestion to point out the content of this paragraph, we have revised the original content according to your suggestion:
 This finding may plausibly reflect an adaptive neuromuscular compensation strategy employed to preserve joint stability in response to asymmetric mechanical demands. Moreover, the non-dominant side, with relatively weaker muscle strength and less precise neural control, may require greater plantarflexion and internal rotation to maintain landing stability [9]. However, the extent to which joint morphology, motor control strategies, and limb dominance contribute to these asymmetries remains unclear and warrants further investigation through integrated biomechanical and neuromechanical analyses.

6. This finding supports Ali et al. [29], revealing a significant increase in knee flexion moment during landing from 45 cm compared to 30 cm.

Thank you for your suggestion to point out the content of this sentence, we have modified the original content according to your suggestion:

This finding supports Ali et al. [3], who reported a significant increase in knee flexion moment during landing from 45 cm compared to 30 cm.

7. To reduce injury risk, rope jumping participants[20]should control landing heights and gradually increase them during training.

Thank you for your suggestion to point out the content of this sentence, we have modified the original content according to your suggestion:

To reduce injury risk, rope jumping participants should consider controlling landing heights and gradually increasing them during training.

8. Thank you for your suggestions on the limitations section of this study, we have revised the original paragraph as follows:

This study, while providing valuable insights, has notable limitations. First, all participants were male, which limits the generalizability of the findings to female populations, who may exhibit different neuromuscular control strategies and joint loading patterns. Future studies should include female participants to explore potential sex-specific biomechanical responses during landing tasks. Second, although the CAST marker placement technique was used to minimize soft tissue artifacts, skin motion artifacts remain an inherent limitation in marker-based motion capture systems and may influence the accuracy of joint kinematic and kinetic measurements. Additionally, the study was conducted under a controlled laboratory setting, which does not fully replicate real-world rope skipping conditions. Moreover, the focus on lower limb cushioning during forward landings overlooks other landing strategies, and the role of upper limb and trunk dynamics remains unexplored. Future research should address these methodological limitations by incorporating more diverse populations, the influence of landing direction and the biomechanical contributions of the upper limbs and trunk, as these factors may significantly affect joint loading and injury risk [33].

9. This study, based on Statistical Parametric Mapping, identified greater hip abduction，…

Thank you for your suggestions regarding the conclusion section of this study, we have modified the original sentence as follows:

This study utilized Statistical Parametric Mapping to analyze landing biomechanics, …

**Thanks again for your valuable suggestions.**

1. [] Kristianslund, E., Krosshaug, T., & Van den Bogert, A. J. (2012). Effect of low pass filtering on joint moments from inverse dynamics: implications for injury prevention. Journal of biomechanics, 45(4), 666-671. <https://doi.org/10.1016/j.jbiomech.2011.12.011>.
2. [] Lenz, A. L., Strobel, M. A., Anderson, A. M., Fial, A. V., MacWilliams, B. A., Krzak, J. J., & Kruger, K. M. (2021). Assignment of local coordinate systems and methods to calculate tibiotalar and subtalar kinematics: a systematic review. Journal of biomechanics, 120, 110344. <https://doi.org/10.1016/j.jbiomech.2021.110344.>
